# Power-Law Scaling in the Brain Surface Electric Potential

**DOI:** 10.1371/journal.pcbi.1000609

**Published:** 2009-12-18

**Authors:** Kai J. Miller, Larry B. Sorensen, Jeffrey G. Ojemann, Marcel den Nijs

**Affiliations:** 1 Department of Physics, University of Washington, Seattle, Washington, United States of America; 2 Department of Neurological Surgery, University of Washington, and Center for Integrative Brain Research, Seattle Children's Research Institute, Seattle, Washington, United States of America; Indiana University, United States of America

## Abstract

Recent studies have identified broadband phenomena in the electric potentials produced by the brain. We report the finding of power-law scaling in these signals using subdural electrocorticographic recordings from the surface of human cortex. The power spectral density (PSD) of the electric potential has the power-law form 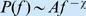 from 80 to 500 Hz. This scaling index, 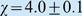, is conserved across subjects, area in the cortex, and local neural activity levels. The shape of the PSD does not change with increases in local cortical activity, but the amplitude, 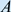, increases. We observe a “knee” in the spectra at 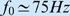, implying the existence of a characteristic time scale 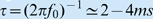. Below 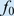, we explore two-power-law forms of the PSD, and demonstrate that there are activity-related fluctuations in the amplitude of a power-law process lying beneath the 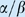 rhythms. Finally, we illustrate through simulation how, small-scale, simplified neuronal models could lead to these power-law observations. This suggests a new paradigm of non-oscillatory “asynchronous,” scale-free, changes in cortical potentials, corresponding to changes in mean population-averaged firing rate, to complement the prevalent “synchronous” rhythm-based paradigm.

## Author Summary

For a very long time, the measurement of the large scale potentials produced by the brain from outside of the head, using electroencephalography and magnetoencephalography, and from inside the head, using electrocorticography, has fixated on changes in specific rhythms and frequency ranges. This fixation presupposes physiologic changes where neuronal populations synchronously oscillate at specific timescales. Here, we demonstrate that there are phenomena which obey a broadband, power-law form extending across the entire frequency domain, with no special timescale. It is shown that, with local brain activity, there is an increase in power across all frequencies, and the power-law shape is conserved. Furthermore, we illustrate through simple simulation how fluctuations in this phenomenon may be linked to increases and decreases in “noise-like” patterns of activity in neuronal populations. Although power-laws have been postulated to exist in background electrical brain activity, the view that local activity can be captured by fluctuations in a broadband power-law in the power spectrum of electric potential timeseries represents a fundamentally new way of thinking about changes in the electric potential produced by the brain, and provides insight into what types of neuronal processes might produce these potentials.

## Introduction

**Figure 1:**
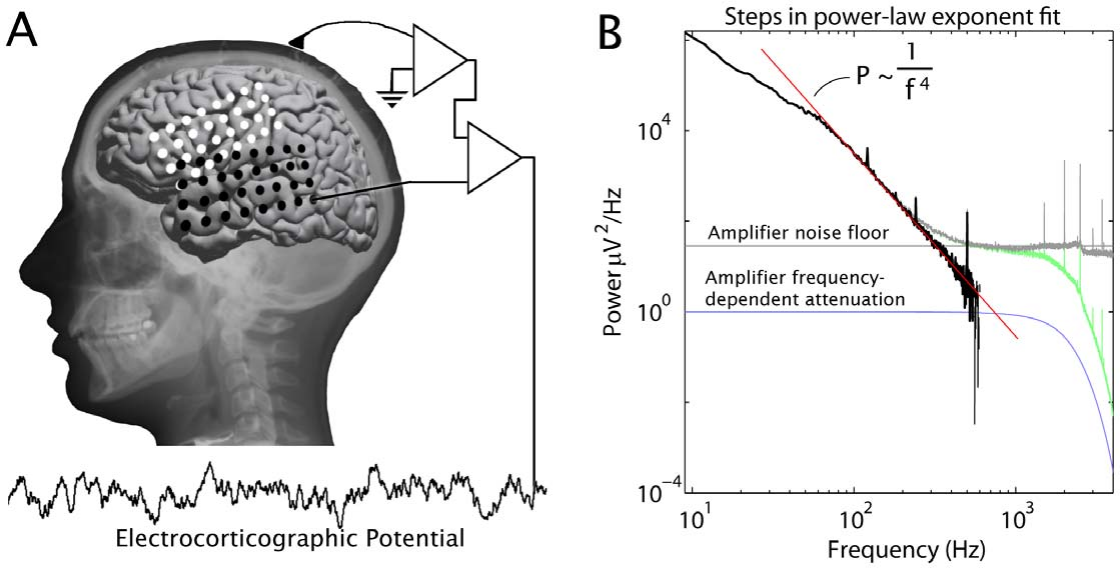
Experimental setting. (A) Cortical surface potential measurement: The electrode array locations are shown on a template brain for subject 1 (black, temporal, positions calculated from x-ray [69]) and subject 2 (white, fronto-parietal). Potentials of all 32 electrodes are measured simultaneously with respect to a scalp reference and ground, before they are pair-wise re-referenced to obtain 52 electrode pair channels. (B) Spectral correction and fitting: The raw power spectral density (PSD) of an electrode channel pair (green) is corrected first for amplifier frequency dependent attenuation (roll-off shown in the blue trace, the corrected PSD is shown gray), and then for amplifier noise floor (denoted by the horizontal black line, with corrected PSD in black). The corrected PSD was then fit linearly on these log-log axes (red line shows fit here), and the slope of this fit determines the exponent of an associated power-law form (here 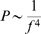). The sharp line noise spikes at 60 Hz and its harmonics were excluded in our fitting analysis (and are thus omitted in Figures 2–4).

Neuronal electrical activity may be measured at many scales, from individual ion channels [1] to the largest scale measurement of electroencephalographic (EEG) potentials entirely outside the head [2]. Synaptic current produces a change in the local electric field, and it is believed that large scale field potentials reveal primarily the aggregate synaptic activity from large neuronal populations [3],[4]. Our particular experiments measure these potentials at the brain surface, using arrays of platinum electrocorticographic (ECoG) electrodes (Figure 1). Interaction properties between synapses, when averaged across the entire ensemble, may be revealed by the potential auto-correlation function:
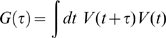
(1)which is an average over the entire time interval of the recording. The Fourier Transform of 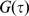 is the power spectral density (PSD), and reveals to what degree the potential at one point in time is correlated with the potential at a later point in time. Because of this, characteristic phenomena in the cortical potential PSD have interpretable implications for the interaction properties between elements within neuronal populations.

For example, since Adolf Beck first described in the 1890s how simple behavioral change produced widespread amplitude changes in rhythmic properties of the electric potential timeseries [5], findings of peaked phenomena in the PSD have pointed to oscillatory activity that is synchronized across the neuronal population [6]–[12], and have been linked to known large-scale brain phenomena like cortical-subcortical feedback loops which change during behavior [8], [13]–[16]. Simple behaviors produce robust change in the oscillations: Opening the eyes decreases the occipital 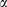-rhythm (8–12Hz) amplitude [17],[18], and movement decreases the lateral frontoparietal 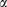 and 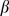 (18–25Hz) rhythm amplitudes [19]–[23].

Other studies have attributed band-specific processes in the so-called “high-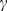
” range (60–150Hz) to local cortical processing [24],[25], with specific timescales linked to the particular choice of frequency range. In lateral brain regions, we observed a lack of distinct peaks in the cortical potential PSD beyond 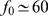
Hz, and hypothesized the existence of broadband changes across all frequencies which were obscured at low frequencies by covariant fluctuations in the 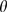/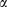/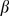 rhythms [22],[26]. While previous studies had hypothesized that background power-laws existed in the PSD [27]–[30], we hypothesized the existence of behaviorally-associated changes in a power-law process of the form 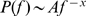, and named attempts to capture it the “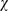-band/index,” at the higher frequencies where it is most plainly observed [22],[26],[31],[32]. Our early studies, sampled at 1kHz, had PSDs that truncated above 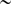
250Hz. Although we observed structure in these PSDs, and hypothesized the existence of a power-law, we needed data with a higher sampling rate to establish it firmly. The purpose of this study was to sample higher, at 10 kHz and determine, as accurately as possible, whether there is indeed such a power-law in the human cortical potential power spectrum, and how it might change with cortical activity. Results from this higher sampling rate (4 subjects) might then allow us to return, informed, to the large group of lower sampling rate data (16 subjects), and re-examine it with knowledge of how it must behave at higher frequencies. Here, we identify and characterize a scale-free process in the ECoG potential PSD, revealed by a power-law. The existence of such a power-law process points to phenomena with no special timescale, where the neuronal population beneath is not synchronously oscillating. We demonstrate through very basic simulation how such spectra might arise from simple processes, and how observed broadband power-law changes in the PSD might simply reflect a change in population mean firing rate.

## Results

**Figure 2 pcbi-1000609-g002:**
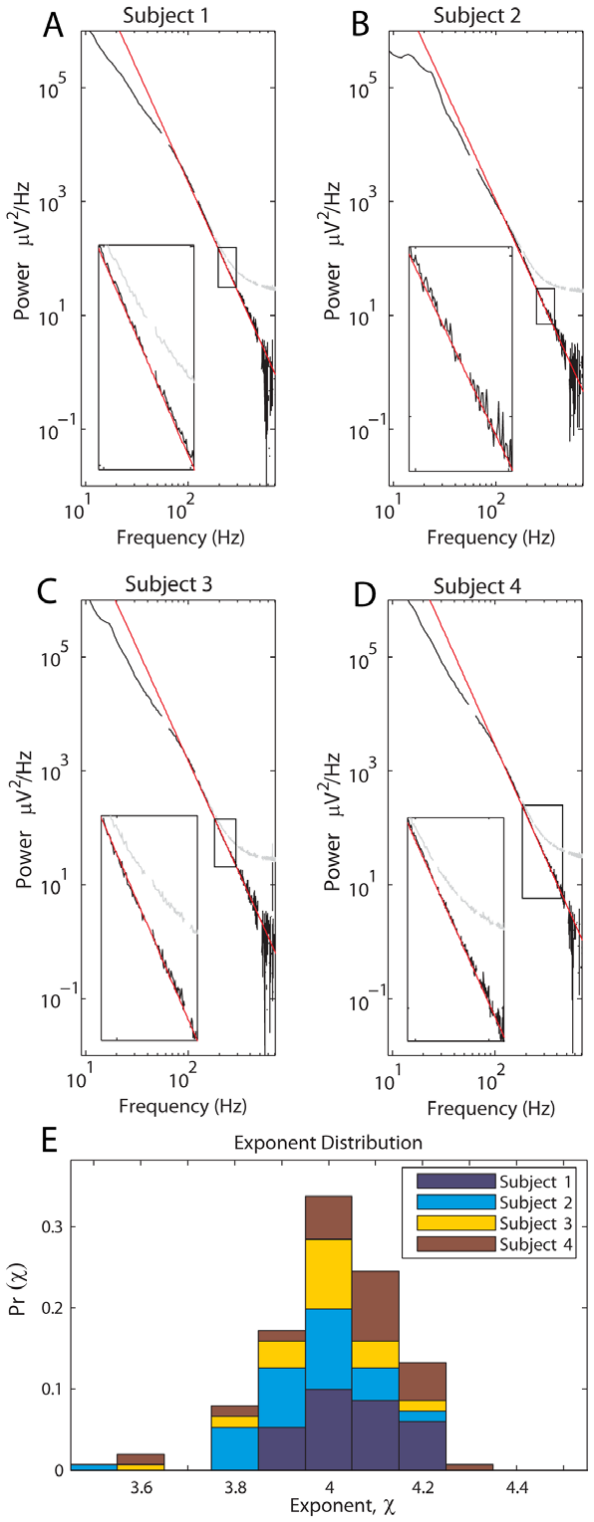
The power-law in the cortical spectrum, above 80Hz. (A) The averaged PSD for all electrode-pair channels from subject 1 (black trace, with 60Hz harmonics omitted; gray line shows PSD prior to noise floor subtraction) on log-log axes, with the red line showing the best linear fit from 80Hz until the PSD hits the noise floor at 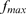 = 579Hz. The blown-up inset illustrates the quality of the fit in the higher frequencies at higher resolution. (B)–(D) As in (A), but for subjects 2, 3, and 4, respectively. The fitting ranges were from 80Hz to 530, 534, 559 Hz for subjects 2–4, respectively. (E) Histogram of the fit exponents of individual electrode pair channels, stacked for subjects 1–4, between 80 and 400Hz.

We measured the surface potential between pairs of surface electrocorticographic electrodes separated by one centimeter from each other on the lateral brain surface of 20 human subjects. From these potentials, we calculated power spectral densities (PSDs) averaged over several minutes of data. As detailed in the methodology section below, each PSD was examined for the presence and character of a power-law form. The strongest empiric finding from this study was the robust fit of a power-law form 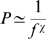, with 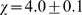, for frequencies above 80Hz. We performed a stringent fitting protocol in the frequency range 80Hz
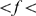
580Hz of the averaged electrode pair PSDs of 4 subjects, and found extremely tight fits to the form 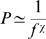 and 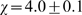 in each case. This was obtained by fitting 10 kHz sampled data from subjects 1–4, during a simple fixation task. Figure 2A–D shows the PSD, averaged over electrode pairs. The inserts illustrate the robust quality of the power-law form, where the jitter of data around the fit is more than one decade down from the signal. The exponent 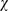 and the parameters 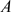 and 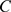 in the form 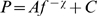 were estimated via a set of log-log least-squares linear fits of the power spectral density, with a range-shrinking scheme to ensure that local fits within the fitting range produced the same fit as the global form. The fitting range chosen for each was a lower bound of 80 Hz (in order to stay above a “knee” at 75Hz) and the highest frequency where spectra could be resolved from the noise floor in each subject (579, 530, 534, 559 Hz for subjects 1–4), excluding harmonics of 60 Hz. The combined spectra fit values of 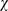 were 3.97, 3.94, 3.97, and 4.02 for subjects 1–4, respectively. The error estimates (of order 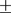
0.1 or less for each subject) were based on robustness against range shrinking as well as the deviations of the best fit with respect to the actual data across the entire frequency range (see insets in Figure 2A–D).

To test for universality, we also performed the same type of fits to each individual electrode pair spectrum for subjects 1–4, from 80 Hz to 400 Hz. The histogram of these individual fits is shown in Figure 2E. The mean of the individual fits was 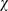 = 4.01, with SD = 0.13 (N = 151), in strong agreement with the averaged spectra. Individual electrode pair channel PSDs, fit between 80Hz
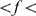
400Hz, produced the same result as the average spectrum, without systematic variation by brain area. This power-law scaling extends over four decades in power and, because 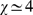 is large, over one decade in frequency. The noise around this straight line is minimal, and is robust against range shrinking (that is, the fit exponent is unchanged if a smaller frequency range interval, within the total range, is chosen for fitting). This quantitative level of power-law scaling is rarely seen in experimental data [33].

**Figure 3 pcbi-1000609-g003:**
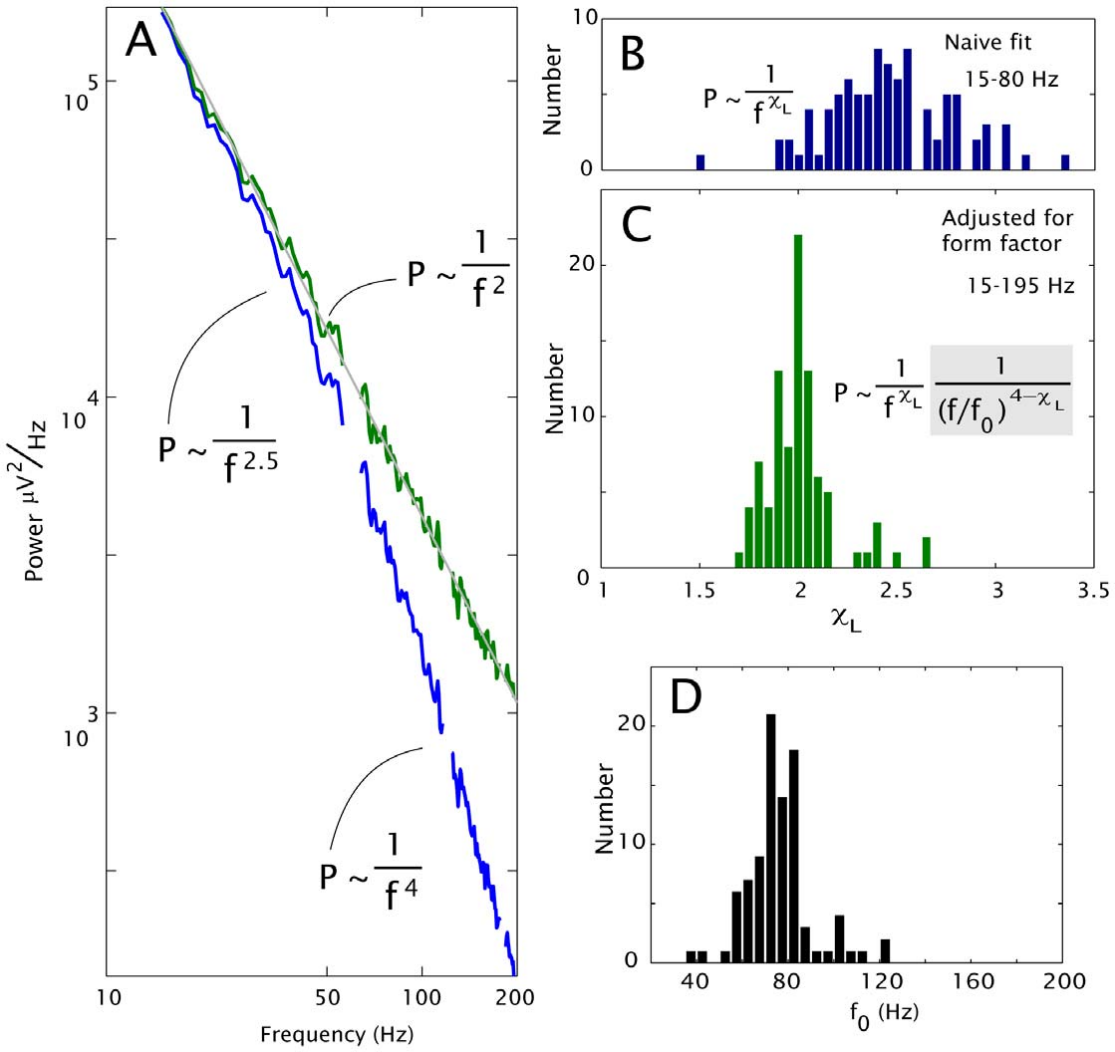
A power-law fit at lower frequency. The data sampled at 1kHz was first fit naively for a low-frequency power law below 80Hz. It was then fit iteratively to identify the corner frequency, 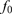, and low-frequency exponent, 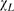, on a case-by-case basis. (A) A single PSD, from subject 19, showing the raw spectrum (blue) with the form 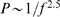 below 80Hz, with a transition to 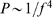 above. After adjusting for a freely-fit Lorentzian form factor 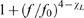, it follows 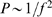 (i.e. 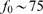 and 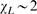). (B) The histogram for naïve fits yielded 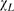 = 2.46
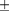
0.32 (mean
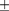
SD, N = 91, fit range 15–80Hz). Fits for a more complex form (equation 2, fit from 15–195Hz), yielded exponent 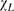 = 2.01
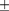
0.18 (in (C)), and corner frequency 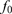 = 77Hz
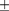
14Hz (in (D)).

Preliminary calculations showed a clear crossover (“knee”) in the PSD at 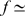
75Hz, below which the PSD takes a different form (see Figures 2 and 3). A natural question to ask is whether there is a different power-law form at lower frequencies, with exponent 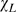. Previous power-law estimates in the cortical potential focused on this lower frequency range [34]–[37], and most naively fit scale-free exponents directly to spectra known to contain scale-dependent phenomena (oscillatory brain rhythms peaked at specific frequencies). We wished to avoid the confounding influence of these rhythms, but, unfortunately, the 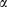
&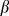 rhythms were strongly pronounced in most cortical channel pairs of the data recorded at 10kHz (subjects 1–4, clearly visible in Figure 2). They obscured whatever asynchronous (scale-free, non-peaked) phenomena might be present underneath at frequencies below 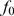, and there were simply not enough channels without them to be meaningfully examined in the 10kHz sampled data. Therefore, we returned to our large set of initial 1kHz sampled data, and circumvented these rhythms with two approaches: the first was simple avoidance, by selection of channels where the rhythms were absent during the fixation task; the second was to use data from an experimental setting (finger movement) which caused the rhythms and the underlying broad-band change to vary differently, and the rhythms could be removed.

In order to evaluate what the exponent in such a lower power-law would be (below 80Hz), we first naively fit the resting spectra of a large ensemble of fixation data (subjects 5–20) sampled at 1kHz. Only channel pairs which lacked 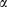
&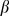 rhythms, and for which the noise floor was relatively small compared with the power, were selected and fit. This naïve fit of a low frequency power law yielded values of 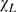 = 2.46
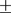
0.32 (mean
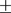
SD, N = 91) (Figure 3). We then modified the power spectra of this 1kHz fixation data, dividing by the product of 2 Lorentzian-like form factors:
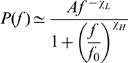
(2)based upon the knee observed in the spectra of the 10 kHz data, subject to the constraint that 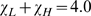 (because the 10kHz fit implied that this must be the case for large 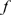), and calculated the values of 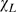 and 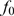 for which the modified spectra had a slope closest to zero, on the frequency interval 15–195Hz. These post-modification fits yielded 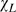 = 2.01
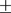
0.18 (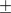
SD, N = 91), and 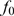 was 77Hz
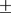
14Hz, as shown in Figure 3. It should be noted that this likely represents a true range, where the “knee” at 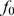 may vary by location and individual.

**Figure 4 pcbi-1000609-g004:**
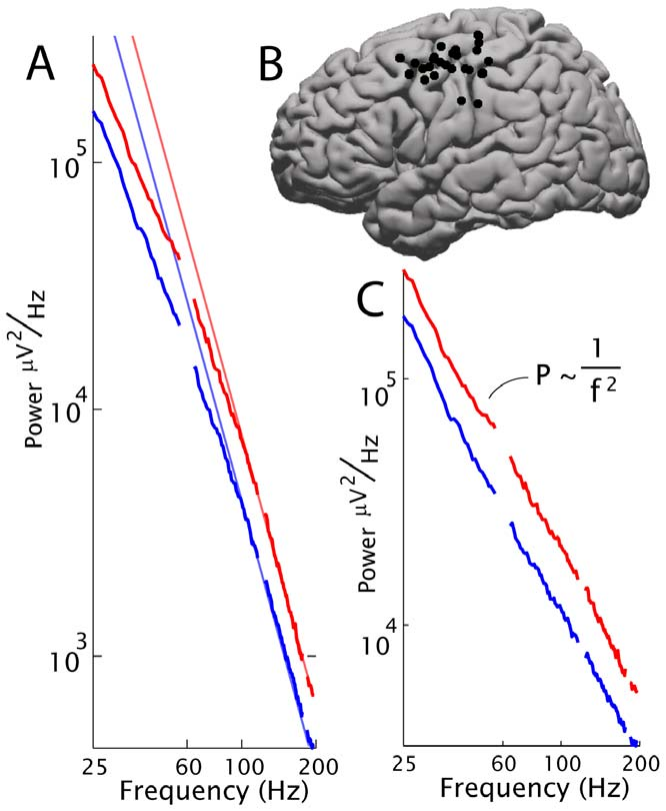
The conserved shape of the power spectrum with brain activity. The average shift in power spectral density in electrodes after removal of the 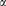/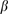 peaks in the hand cortical area during finger movement, for subjects 16–20; 5 electrode-pair channels each (25 total). (A) This shift demonstrates that, in motor cortex, movement (red) increases the overall power while preserving the shape of the rest (blue) spectrum. 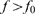 power-law fits (grey) are consistent with 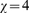. (B) Channel locations (interpolated between the two paired electrodes) across all subjects, projected to the left-hand side. (C) Remaining spectra after dividing out a Lorentzian, 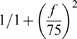 to illustrate consistency with the two-power-laws form with 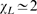. The residual lines are consistently parallel, and therefore, the exponent shift with activity in individual channels is negligible (shift in exponent  = 0.03
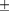
0.09, 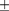
SD, N = 25, 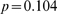, by paired t-test). The shift up is an overall factor of 1.76 with a variation (standard deviation) of order 0.31 (maximum 2.47, minimum 1.29, N = 25, 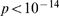).

The change in the shape of the PSD 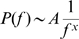 during different levels of neuronal population activity reveals different dynamics within the population. A shift in the exponent, 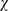, would suggest a change in the correlation between neurons, whereas a shift in the coefficient, 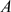, would suggest an overall increase or decrease in population activity. In a recent manuscript [38], we demonstrated how motor-behavior-related variation in the 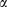 and 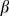 bands allow them to be removed from the measured PSD in primary motor cortex. We repeated the same process as that on pair-wise re-referenced electrode channels and, removed the oscillatory (peaked) phenomena from the PSDs. Activity-related changes in individual channel pairs were examined by dividing the active, movement, spectra (“
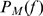
”) by the inactive, rest, spectra (“
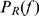
”), element-wise (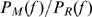). Calculating the 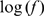 vs. 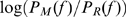 slope removes the common shape (including the effect of 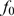), and reveals whether there is a shift in the slope, 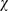, during activity. This shift in exponent, when fit from 25–195Hz, was insignificant: 0.03
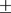
0.09, (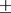
SD, N = 25, 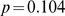, by paired t-test; subjects 16–20, 5 electrode pairs from motor cortex each). Because there was no significant shift in the exponent, then active/inactive power ratio 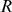 (between the amplitudes 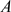, Figure 4) was obtained for each channel simply by averaging 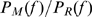 across frequencies in each channel. The geometric mean of these ratios was 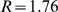 with a variation (standard deviation) of order 0.31 (maximum 2.47, minimum 1.29, N = 25, 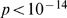).

**Figure 5:**
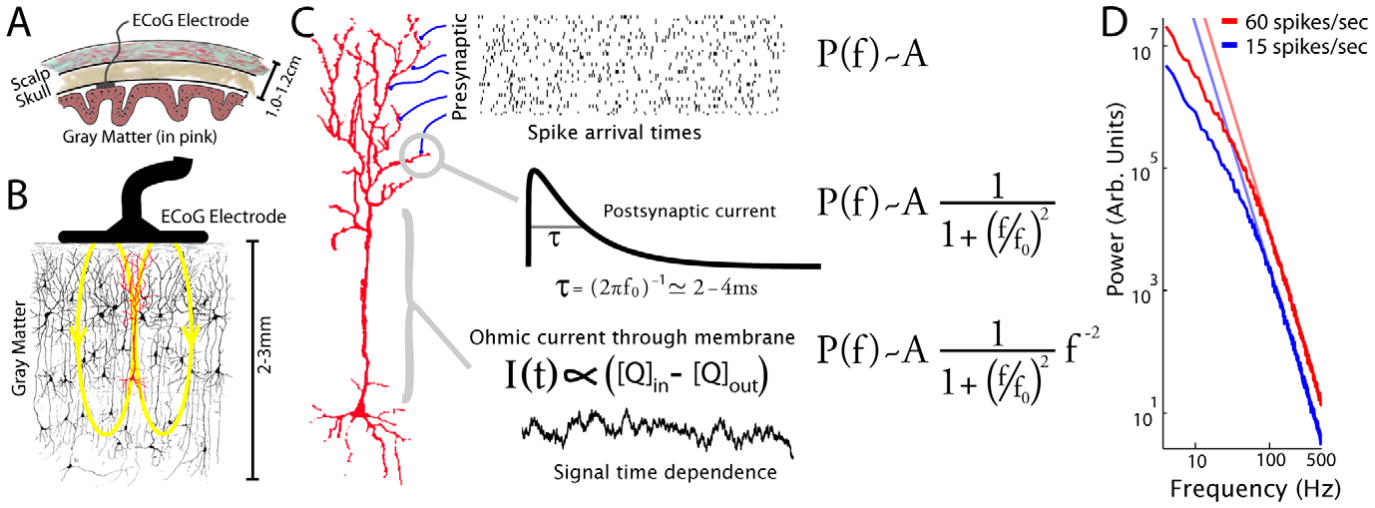
How these power-law phenomena in the cortical potentials might be generated. (A)–(B) Beneath each one of our 2.3 mm diameter electrodes, there are of order 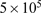 neurons, and each of these has 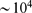 synapses where it receives input [39]. Currents into and out of each of these 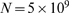 synapses, and gradual ohmic current through the dendritic membrane as the post-synaptic charge bolus diffuses toward the soma, produce transient dendritic dipoles that are thought to be the source of the macroscale potentials, 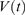, that we measure with ECoG. (cross-sectional stain from Ramon y Cajal) (C) In our simple simulation, a single neuron receives 6000 presynaptic inputs. Each of these inputs delivers a temporally Poisson-distributed series of action potentials (with each arrival time as a delta function). Each of these action potentials induces a post-synaptic current shape which has an extremely fast rise and an exponential decay. The input from all synapses is combined, and charge accumulates over time and is lost, ohmically, through the dendritic membrane. We approximate the time dependence of the current dipole that produces our potentials as the time dependence of this ohmic membrane current (bottom trace in C). The equations to the right of the schematic indicate how each element of this heuristic illustration would contribute to the experimentally-fit analytic structure of the PSD. (D) The PSD of this signal has a knee at 70Hz, with power-law of the form 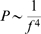 beyond. The change in the spectra with increasing mean spike rate of synaptic input strongly resembles the change observed experimentally over motor cortex during activity (Figure 4).

A simple simulation using Poisson-distributed pre-synaptic action potentials to a single neuron beneath one of our electrodes (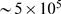 neurons beneath each [39]) reproduced the spectra that we see, at different levels of cortical activity with simulated rates of 15, 30, and 60 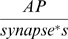 (Figure 5; “
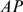
” = action potential). Using a modified leaky integrate-and-fire model (without a firing component), consisting of an exponentially decaying post-synaptic current, temporal integration, and passive current efflux to estimate the time-dependence of a dendritic current-dipole field, a spectrum with the power-law form 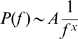 emerged. A 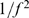 factor, contributed by exponentially decaying synaptic current, directly follows previous work by Bedard et al [27]. The value of 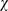 for high frequencies (80–500Hz) in this simulated data was 4.0 at all three levels of cortical activity (linear fit on log-log axes). The ratio of the coefficient, 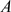, was 4.03 for 60 vs. 15 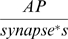, and 1.96 for 30 vs. 15 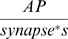. Because the PSD of the superposition of many such uncorrelated model neurons will have the same 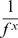 shape as one, the model in a single neuron will generalize to an entire neuronal population.

## Discussion

Although traditional EEG studies have been limited to measurements of frequencies below 100Hz, the timing of fundamental neuronal processes suggests that information content should be present at much higher frequencies. Propagation time of a spike along an axon, synaptic neurotransmitter diffusion time, or the recursion time of reciprocally coupled neurons are all near or below 10 ms [39]–[42]. Synchronizations and correlations associated with these should exist at least up to 1kHz.

The human brain is arguably the most complex and largest network available to observe scale free behavior in a natural setting, and at the 5mm^2^ scale of our electrodes, we have observed robust power-law scaling. Power-laws represent scale free behavior – the finding of which typically evokes discussion of scale free networks, complexity, avalanches, and self-organized criticality (SOC) [43]–[45], and if our measured value of 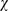 were distinct from an integer, we might have discussed SOC at the cortical surface. SOC is a process where very complex global phenomena arise in a population of interacting elements due to very simple properties of the individual element, and simple rules that dictate how pairs of elements interact with one another [43]. The global complexity is generally not immediately apparent from the simple properties and rules. A popular example of this is the emergence of earthquakes of different sizes and the frequencies on which they occur [46], which have a power-law relationship. The form of this earthquake distribution that is observed in nature can also be derived from a simple model where blocks of matter are held together by springs, but slip against one another with friction [47],[48] – global complexity emerges from basic interaction, and can be characterized by a power law. One might hypothesize that the interaction between neurons in a population, producing sophisticated computation, would exhibit SOC, and be revealed by a power law. Our experimental finding of power law scaling in the brain surface electric potential does not suggest SOC. Non-integer exponents in power-law relationships imply self-organized criticality in the population of constituent elements, but multiple-of-two integers, such as our finding of 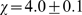, point towards simple, noise-like, analytic functions, i.e. to a non-singular, non-fractal, non-complexity explanation of the shape of the power spectrum (such as a diffusive process or filtered noise). Perhaps SOC behavior (if it exists) is only expressed in the PSD in more subtle ways, within the 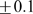 uncertainty, or at finer spatial scales, in the cortical surface potential. While not present in this study, evidence for complex neural correlations (and different exponents) may emerge in different experimental settings, such as power-fluctuations in the 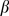-rhythm [49], in the magnitude of spatially-correlated cascades of activity in the LFP [50], or the gain of neuronal firing in response to cyclical driving potentials [51].

In order to examine the PSD structure at lower frequencies, the 91 channels from subjects 5–20 without 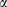
&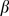 rhythms were first blindly fit linearly on log-log axes up to the knee in the spectrum, from 15–80Hz, producing an estimate of a low-frequency power-law with exponent 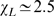. Based upon the knee at 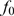, and the blind fit in Figure 3a, we examined a more complex parameterized form of the PSD which accounts for the knee, could be fit across a larger range (15–195Hz), and goes to 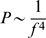 for large 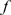 (i.e. of the form of equation 2). Note that the discovered factor of 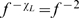 might represent a form factor 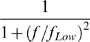 where the lower boundary of our fitting range is above 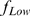, so a lower knee with a flattening of the spectrum is not appreciated. There may, in fact, be a lower “flattening” of the spectrum, below 20Hz, that is masked by the 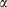 and (ubiquitous) 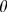/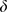 rhythms (see, for example, plots in [27],[28]). As described by Sigeti and Horsthemke, these types of “2+2” spectra can emerge from noise-like processes which have two simple correlation times [52]. There are many such combinations of two simple known neuronal processes, such as temporal integration in dendrites or soma, exponentially decaying membrane currents, low-pass RC filtering by tissue, or local network connectivity which, when modeled, will produce precisely this form (one such is illustrated in Figure 5).

In a previous paper [22], we hypothesized that observed high frequency changes called “high-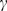
” [53] or 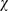 [31] were reflective of broad-band, power-law shifts, and were obscured by the 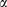
&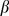 rhythms at lower frequencies in motor cortex. Indeed, the intersection of these two phenomena, “
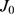
”, was subsequently shown to lie at 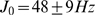 (mean
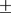
SD) (range 32–57 Hz) during hand motor movement [26]. When we made this hypothesis, it was uncertain whether this shift might reflect a change in the exponent, 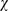, or the coefficient, 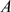, of a power-law of the form 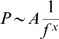. In a more recent manuscript, we performed a decomposition technique which removed the 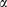
&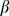 rhythms [38], revealing broadband increase beneath. Figure 4 shows that when this method is applied, and the residual broadband spectra are modified with a Lorentzian form, both active and inactive spectra are approximately linear on a log-log plot with slope −2. In other words, they can be reasonably described by a power-law with exponent 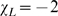. When individual channel pairs were examined independently for active and inactive spectra, there was no difference in fit exponent: the shape of the PSD was unchanged, but the overall amplitude was. This implies that, at the spatial scale of our electrodes, after spatially and temporally averaging, the structure and complexity of the large-scale neural networks do not change during computation, but the overall amount of activity does. The active/inactive power ratio between the amplitudes (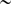
1.76) provides a sense of the dynamic range of this network in the behaving brain as it shifts between ‘idling’ and ‘computing’ regimes.

An important caveat to these findings is that the PSDs which we fit were averaged over long periods of time (minutes of fixation or seconds of movement/rest). If the same is done over very small windows, there are deviations from the form of the averaged PSD. It is within these small windows that computations take place, and the “instantaneous PSD” will not have the power-law shape at all times. Without reoccurring synchronized oscillatory processes, however, it averages to the power-law shape over time.

To gain intuition about what may produce these signals, we performed a simple simulation from the perspective of a single neuron beneath one of our electrodes (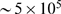 neurons immediately underneath each electrode [39]), and take into account only three factors: Poisson-distributed input action potentials, exponentially decaying post-synaptic currents, and ohmic current in the dendrite, produced a time-dependent signal with a PSD of the same shape that we measure, and with the same change during increase in activity. While our particular choice of model was one of many potential models, we believe that any simulation of the ECoG PSD should rely on very simple factors, ubiquitous in the cortical neuronal population, because the effect must be conserved after averaging across 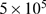 neurons. Although this simulation was largely oblivious both to the details of dendritic and overall neuronal processing (between neurons, etc), and to many factors which must influence the creation of dendritic current dipoles, it does exhibit two things that we would like to stress. The first is that the knee we observe in the spectra likely corresponds to the timescale of a very simple process, like post-synaptic potential current of particular timescale, which occurs throughout the cortical surface. The second is that changes in the amplitude, 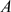, of the power-law reflect changes in Poisson-distributed (after coarse graining) input action potentials beneath each of the electrodes. Indeed, we have recently shown that the capture of this broad-band, here demonstrated to obey a power-law, reveals local cortical activity with high temporal precision [38]. The values that the simulation obtains (factor of 2/4 increase in 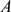 with a doubling/quadrupling of the action potential rate) suggest that the difference we observe experimentally during finger movement might represent roughly a doubling of mean input action potential rate for the population of neurons.

Collectively, these findings have important implications for understanding the electric potential at the cortical surface, with the necessary caveat that the effects seen reflect an average of 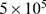 neurons. Because of the connection between the autocorrelation in the potential and the PSD (equation 1), we can try to connect the form we observe in the PSD to correlation in the physiologic processes which produce it. At this coarse level, there is a special frequency at roughly 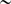
75Hz, and this must be accounted for. This may be due to an exponentially decaying temporal correlation of 2–4ms from post-synaptic current, tissue low-pass filter, protein dissociation, or some other. Perhaps this timescale corresponds to a recurrent process of 11–16ms such as characteristic reciprocal connectivity in local neuronal circuits, or to the “conduction time” of single neurons – how much time it takes for a coordinated pre-synaptic super-threshold pulse to produce an action potential at the axon hillock. If there is a lower “knee” below our fitting range, and masked by the theta rhythm, that implies a second timescale with physiologic importance of its own which must be accounted for. Each of these must also correspond to a factor of 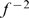 above the associated characteristic frequency. Our simple simulation follows that of Bedard et. al. [27] for the first factor, with exponentially decaying post-synaptic current accounting for 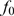 and one factor of 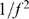, and charge accumulation in the dendrite producing the second factor of 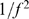, with the dendritic current leakage producing a second native frequency well below any fitting range we examined. Fluctuations in firing rate produce overall increases and decreases in the PSD, without a change in the frequency dependence of the PSD.

Our experimental results, in contrast, differ significantly from the Bedard et. al. paper [27]. They reported a 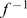 to 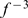 transition in their PSD measurements. As we do, they attribute a factor of 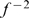 to Poisson-distributed spikes and the shape of the post-synaptic current. They attribute the remaining 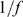 to passive tissue filtering, which has since been contradicted experimentally by Logothetis et. al. [54]. To the eye, the PSD from the Bedard et. al, study appears as if it may have been better fit by an 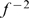 to 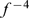 shape like the one found in this study. By extension of their logic to our finding, our power-of-two structure may point away from the presence of 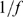 tissue attenuation.

Activity-related narrow-band PSD increases, correlating with fMRI [55], have been demonstrated in the “high-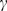
” (40–100Hz) frequency range of the LFP [56]–[60] and the MEG [61],[62]. In each case, these are peaked phenomena in the PSD, reflecting a coherent, oscillatory process, which increases with activity, and is specific to visual cortex. This is a very different phenomenon from the power-law increase that we demonstrate here. In fact, Siegel and Konig, in 2003, explicitly distinguished between a peaked, lower 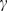, phenomena at 44–53 Hz, and a different, broad-spectral increase, beginning at 45 Hz extending well beyond 100Hz to the upper limit of their recording from cat visual cortex [57]. Henrie and Shapley, as well as Liu and Newsome, made the same distinction, with similar effect, in visual areas of the non-human primate [58],[60]. Extracted broadband changes across the entire human ECoG spectrum, after removing the low-frequency rhythms, were recently demonstrated to capture the timing of individual finger movements with very high fidelity, and explicitly better than band-filtered high-frequency changes [38]. Even more recently, broadband LFP changes were demonstrated to correlate more highly with mean firing rate than any particular frequency band in single unit recordings from human cortex [63]. We suggest that what these manuscripts identify as broadband change, distinct from 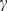 oscillations, and what others have called “high-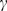
” when referring to broad spectral increases [24],[25],[42],[53], are primarily shifts in the noise-like process identified here, captured at frequencies above the range of band-limited oscillations. This power law process likely reflects the mean input spike rate to neuronal populations, without a preferred timescale. True 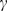-oscillation, however, is likely due to population synchronization by fast-spiking inhibitory interneurons [64],[65], reflected by peaked elements in the PSD, and possibly specific to visual cortex (note that none of the data in this manuscript was recorded from occipital visual areas).

When one is sitting on the seashore, it is possible to hear individual waves breaking, first on the left, and then on the right, correlated by their relation to shape of the shore. As one walks away, however, the correlation between individual waves is lost because many are heard at once, from progressively larger stretches of the beach. The combination of our empirical and modeling findings point to a similar picture, where the internal correlations between neuronal events are lost by averaging over large spatial areas, but the changes that we measure do inform us about the overall number of events taking place in the population.

We would like to propose that the popular “high-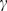
” range, where it has been postulated that synchronous, rhythmic, action potential activity produces changes, is often a reflection of changes in asynchronous activity instead, and revealed by this power-law process. This shift in thinking, to noise-like non-oscillatory changes, is a fundamentally new addition to the way people think about changes in the cortical potential spectrum. Whereas changes in characteristic brain rhythms are thought to reflect synchronized populations that coherently oscillate across large cortical regions, power-law scaling likely reflects asynchronous, averaged, input to the local neural population.

## Materials and Methods

In addition this section, there is an extensive methodological supplement (Text S1), addressing each aspect of the analysis in further detail, with illustration, for the more involved reader.

### Ethics statement

All patients participated in a purely voluntary manner, after providing informed consent, under a protocol approved by the Institutional Review Board of the University of Washington

### Experimental setting

Twenty epileptic human subjects had subdural electrode arrays placed on the brain surface of the lateral frontal, temporal, and parietal cortical areas for the localization of seizure foci. These arrays were composed of circular platinum electrodes with 2.3mm diameter exposed, at 1 cm inter-electrode distance (center-to-center), embedded in silastic. Electrodes lying on top of vasculature, near seizure foci, or with aberrantly high noise floors were excluded from the study. All kept data were recorded away from seizure times. Potentials at each electrode were recorded at 10 kHz (subjects 1–4) or 1 kHz (subjects 5–20). The first type of experiment, fixation, was performed (subjects 1 to 20) by the subjects fixating with their eyes open on an “X”, on the wall 3m away, for several minutes. The second type of experiment (subjects 16 to 20) consisted of simple repeated finger movement (visually cued).

### Spectral calculation

The brain surface potentials from the array were first re-referenced in terms of neighboring differential pair channels (bipolar re-referencing for all nearest neighbors), which significantly reduced the overall noise in the signal.

#### Fixation spectra

The potential time series were broken-up into 1s epochs, overlapping by 0.5s. Each was Hann-windowed, Fourier transformed. The average over epochs was the power spectral density (PSD).

#### Movement spectra

For 5 hand region channels per subject, one second epochs flanking time of maximum flexion of each finger movement (dataglove 5dt, Irvine, CA)), and random one second epochs during times of no movement were identified, Hann-windowed, Fourier transformed, and absolute squared. A principal component method was used to remove the 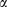
&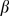 rhythms [38], and the resulting spectra were averaged for movement and rest separately.

### Correction for amplifier artifact

We empirically determined the amplitude attenuation function of the amplifiers independently using an external function generator. For the 10kHz data, a “reasonable range” of empirical amplifier noise floors was determined experimentally by measuring the potential across an equivalent conformation of resistors. Because there was a range of potential floor values for each electrode, depending upon the day, temperature, room, etc, the specific noise floor subtracted from each calculated spectra was determined within the empiric range using a recursive, self-consistent method.

### Spectral fitting of the 10kHz fixation data

For the 10kHz sampled, fixation task data, the value of the exponent 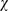 in the power-law relation 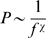 was obtained by fitting a straight line to the experimentally measured PSD, 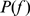, on 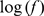 vs. 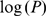 axes, after correcting for amplifier imposed artifacts (Figures 1–5). An infamous mistake in this procedure is to apply global least squares fit, and leave it at that. On a log-log plot, that assigns too much weight to the highest density of datapoints, at high frequency, where the low power and high relative influence of the noise floor make the data noisiest. In reality, a fit should be stable throughout the fitting range, and we employed a technique which is robust against range shrinking to a sub-range within the total fitting range. We determined local fits for the exponent 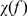 by performing least-squares linear fits to the power spectrum (on log frequency by log Power axes) to obtain local slopes over varying frequency intervals, 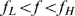 (harmonics of 60Hz were explicitly excluded). The most appropriate value of 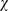 globally is the one that is most stable across many values of 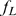 and 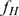, for a given value of the noise floor, 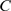. Because variation in the noise floor exists and confounds the quantitative analysis, the appropriate value in a given channel pair for a given experiment is a self-consistent, recursive, 3 parameter fit to the form 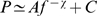, over the entire frequency range 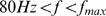, treating 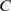, 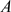, and 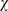 as free fitting parameters (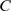 is constrained within the empiric range of experimentally measured noise floors). In each iteration, 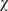 is determined as the average exponent from a distribution of fit exponents, each calculated from a different sub-interval 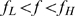. Lower fit values ranged from 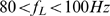 and higher fits ranged from 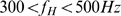. The smallest value of 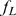, 80Hz, was chosen so that it would be sufficiently above an apparent “knee” in the PSD at 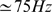. The highest value of 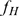 was dictated by the noise floor (beyond which the amplitude of the signal was far below the amplitude of the noise).

### One-kHz fixation data and lower frequency fit

After rejecting electrode pair channels which had notable 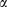 and 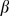 peaks in the PSD or a high noise floor (leaving 91 channels), the 1kHz fixation data from subjects 5–20 was fit to a power law form below 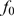. They were corrected for frequency-dependent amplifier attenuation, but not for noise floor, since the contribution of the noise floor to the spectra was not pronounced in the fit range chosen, or the channel was rejected if an excessive noise floor was observed.

First, a naïve linear least-squares fit was performed between 15–80Hz on the plot of the PSD the on 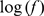 vs. 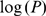 axes for each electrode-pair channel independently. We then divided the PSD through at each frequency by the form of equation 2, and, based upon the fit of 10kHz data, set the constraint 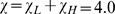 (i.e. for 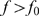, the form goes to 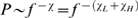, so 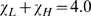). We then iteratively fit 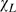 and 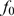, until both converged on stable values. Each iteration consisted of two steps. The PSD is first multiplied by a factor of 
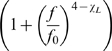
, and the residual is fit on a log-log plot between 15–195Hz to determine a new value of 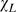. Then, the PSD is divided by the full form of equation 2, for all values of 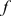 15–195Hz in 0.25Hz increments, and the residual is fit on a log-log plot; the value of 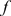 for which the slope of the fit is closest to zero is chosen as the new 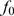. These two steps were iterated until both 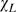 and 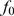 converged to stable values.

### Spectral changes in active cortex

A finger movement task was used first as a tool to first remove synchronous rhythms from the PSD and then examine changes in the PSD during increases in brain activity. A method developed in a recent manuscript characterized how differing covariance between frequencies during different tasks allows underlying motifs to be isolated from the PSD [38]; we removed the motifs corresponding to the low frequency 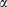/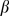 rhythms during a finger movement task. Because this was shown to remove most, but not all of those rhythms, we avoided the center frequency of the beta rhythm, and performed fits to the data above 25Hz; without this method, we would not be able to address shifts in the power law process below 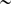
60Hz, where the beta rhythm causes an intersection between the movement and rest spectra [26]. The residual movement PSD 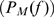 was then divided by the rest PSD 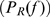 at each frequency. A least-squares linear fit was then performed on the plot of 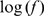 vs. 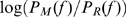. The slope of this fit for each channel pair reveals whether there was a shift in the exponent, 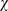, of the power-law shape spectrum 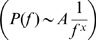 in “active” cortex. Because the shift in slope was found to be zero, then the frequency-averaged ratio 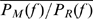 reveals the relative overall shift in the coefficient, 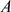. The significance of this shift in 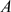 was estimated with a t-test of the distribution of 
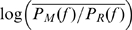
 vs. zero (across electrode pair channels).

### Simulation

While there are many potential models that are mathematically consistent with the form of the spectrum that we found experimentally, we performed a simplified simulation of only one such model. We model one pyramidal neuron with 6000 synapses, and only 3 simple processes to produce time dependence: Poisson-distribution arrival times of pre-synaptic action potentials (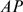, timeseries denoted 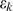 below); stereotyped, transient, exponentially decaying post-synaptic current, with 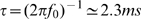 (consistent with experiment [40], 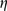 below), and each synapse has random peak current on the interval from −1 to 1 (arbitrary units, 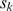 below). These are summated, and integrated over time (representing accumulated charge [66]). The leakage current of this charge through the dendritic membrane, produced by a transmembrane potential, is what we simulate as the time-dependence of the dendritic dipole (with time constant 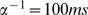 [67]).
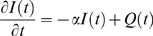
(3)

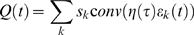
(4)


Where we denote a series of delta functions reflecting the spike arrival times at synapse 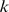 as 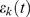, the shape of the post-synaptic response as 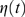 (total length 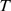), a random number on the interval from −1 to 1, as 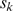, the decay timescale for dendritic current efflux as 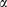, and the convolution operation 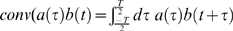 (and 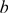 is zero padded at the edges). 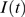 is the simulated time dependence of our surface potential measurements, from which we calculate our simulated spectrum.

As noted by Sigeti and Horsthemke [52], “2+2” spectra, such as the one we have observed from cortical surface spectra, can result from linear systems described by:
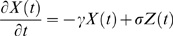
(5)

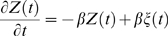
(6)


Where 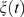 is a Gaussian white noise variable, and the resulting power spectrum of 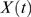, 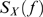, has characteristic corner frequencies at 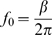 and 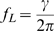. Note that equation 6 is the same as equation 4, but that we explicitly construct 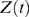 with a convolution that allows us to connect the expression to known physiology in an intuitive way (but 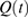 and 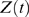 ultimately do have the same properties, where the decay timescale of our 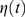 is 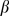).

Our representation is intended to make the connection between the simulation and simple small-scale physiology more intuitive. The timing of this ohmic transmembrane current produced by accumulated charge gradient across the dendritic membrane is modeled as the time dependence producing the macroscale PSD. 6000 synaptic inputs, with input firing rates of 15, 30, and 60 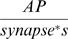 were simulated for 2 minutes of 10kHz data [39]. Recent in-vivo simultaneous transmembrane and local field potential recordings have demonstrated a strong correlation between these two [68], suggesting that models like this, based upon a relationship between post-synaptic potentials and field potential, may provide useful insight.

## Supporting Information

Text S1 Supplemental Detailed Methodology
(9.64 MB PDF)
