# Supplementary material for: Power-Law Scaling in the Brain Surface Electric Potential
Source: PLoS Comput Biol. 2009 Dec 18;5(12):e1000609. doi: 10.1371/journal.pcbi.1000609 (PMC2787015; doi:10.1371/journal.pcbi.1000609)
Supplement: Text S1 — Supplemental Detailed Methodology (9.64 MB PDF) [file pcbi.1000609.s001.pdf]

Supplemental Material for the Manuscript:
Power-Law Scaling in the Brain Surface Electric Potential

Kai J. Miller[1], Larry B. Sorensen[1], Jeffrey G. Ojemann[2], Marcel den Nijs[1]
Departments of Physics[1] and Neurological Surgery[2] ,
University of Washington, Seattle, Washington 98195, U.S.A.
(Dated: October 27, 2009)

This is a supplement for the paper titled "Power-Law Scaling in the Brain Surface Electric Potential". In this supplement we present details of the experimental set up and analysis: amplifier characterization (roll-off and noise floor). We discuss details of the data handing and power law analysis steps. At the end, we also introduce the simulation in more detail.

I. EXPERIMENTAL METHODS

A. Subjects

Subject	Age	Sex	Hand	Grid Location	Seizure Focus
Fixation (recorded at 10 kHZ)					
**1**	23	M	R	R Frontotemporal	R Temporal
**2**	42	M	R	L Frontotemporal	L Temporal
**3**	33	F	R	R Frontotemporal	R Temporal and Orbitofrontal
**4**	18	F	R	L Frontoparietal	L Frontal
Fixation (recorded at 1 kHZ)					
**5**	31	F	R	R Frontotemporal	R Insula
**6**	31	M	R	R Frontotemporal	R Temporal
**7**	21	M	R	R Frontotemporal	R Frontal
**8**	49	F	R	R Temporal-Parietal	R Temporal
**9**	23	M	R	L Frontotemporal	L Temporal
**10**	24	M	R	L Anterior Frontal and Temporal	L Anterior Frontal
**11**	24	F	R	R Frontotemporal	R Orbitofrontal and Temporal
**12**	41	M	L	L Frontotemporal	L Temporal
**13**	45	F	R	L Frontotemporal	L Frontal
**14**	38	M	R	R Frontal	R Frontal
**15**	39	F	R	R Frontal	R Frontal
Both Fixation and finger movement (recorded at 1 kHZ)					
**16**	19	F	R	R Fronto-parietal	R Parietal
**17**	18	F	R	L Frontal	L Frontal
**18**	35	F	R	L Frontotemporal	L Temporal
**19**	32	M	R	L Frontotemporal-parietal	L Temporal
**20**	27	F	R	L Frontotemporal-parietal	L Frontal

Figure S1: Subjects who participated in the study

Twenty-one human subjects (ages 18-45, 8 females), see table in Fig.1, were implanted with subdural electrode arrays for the localization of seizure foci prior to surgical treatment of medically refractory epilepsy. The arrays were typically placed for 5-7 days with the location of the electrodes and duration of implantation determined independently by clinical criteria alone. Experiments were performed at Harborview hospital at the University of Washington (UW). Subjects were typically studied 4-6 days after craniotomy and electrode placement to allow for recovery from the surgery. Subjects gave informed consent for participation in a manner approved by the Institutional Review Board of the University of Washington.

B. Recordings

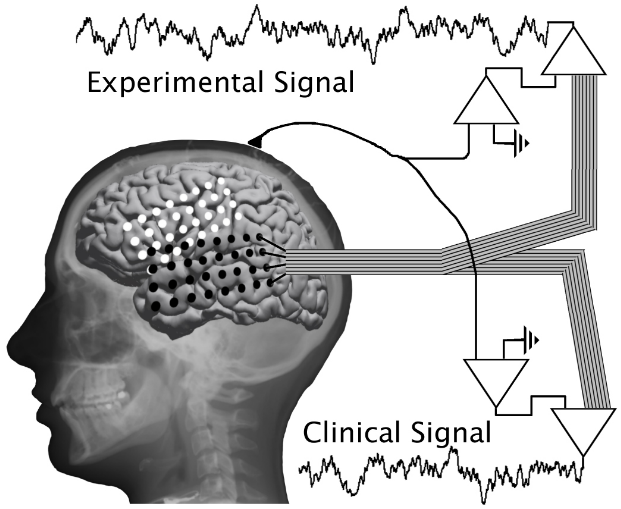

Figure S2: Recording Setup with clinical and experimental amplifiers recording in parallel (with scalp reference).

All data were recorded at the bedside with Neuroscan Synamps^2 amplifiers (Compumedics-Neuroscan, San Antonio, TX), in parallel with a clinical recording system (XLTEK or Nicolet-BMSI), as shown schematically in Fig. 2. The signal was split outside of the head, prior to amplification. The two amplifiers used common ground and reference (both from the scalp).

The platinum electrodes (Ad-Tech, Racine, WI) were configured as 8x{4,5,6,8} rectangular arrays. The elec-

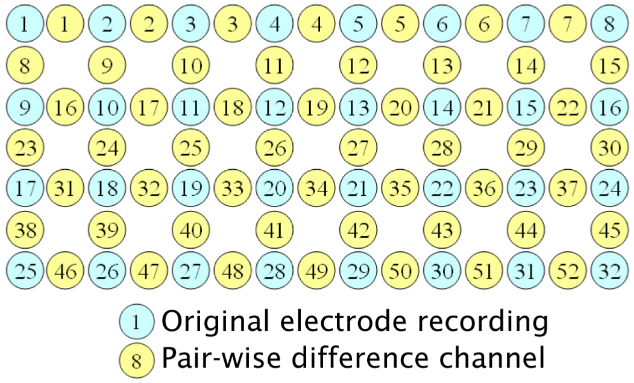

Figure S3: 52 nearest-neighbor differential pairs were generated from the original 32 electrode grids

trodes were 4mm in diameter (2.3mm exposed), at 1 cm inter-electrode distance, and embedded in silastic The arrays were placed on the lateral frontal, temporal, and parietal cortex - shown for subjects 1 (S1 - black) and 2 (S2 - white) on a template brain in figure 2 and on the actual brains in figure S4 for subjects 1-4. Because vasculature significantly changed the nature of the signal, electrodes which rested on top of vasculature were excluded from analysis. Operative photographs showing locations of this rejection are shown in figure S4. For the 4 subjects sampled at 10kHz, the 32 electrodes initially chosen (before rejecting based upon vasculature) were from the portion of the 64 electrode grid away from the seizure focus. For the other 16 subjects, we simply avoided seizure focus sites.

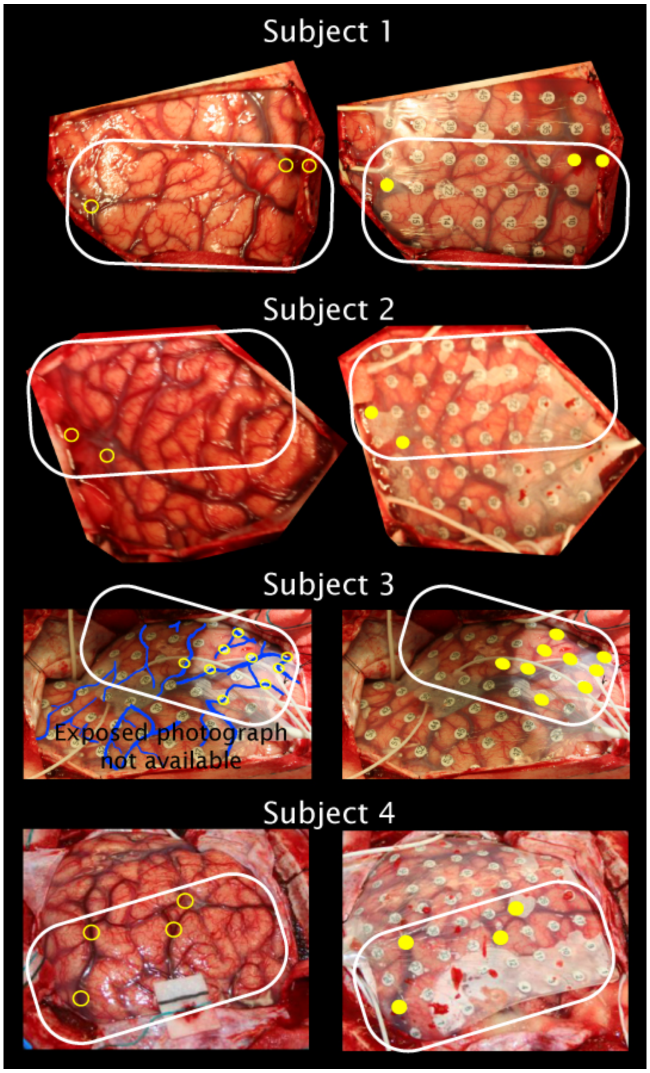

Figure S4: Electrode array placement on cortex for subjects 1-4. The yellow dots indicate electrodes which were rejected for analysis based upon their location above vasculature. The white boxes enclose corresponding cortical areas on exposed photographs (left) and photographs with grids in situ (right).

C. Tasks

All experiments were performed at the bedside.

1. *Fixation*

The subjects fixated on a 10cm "×", on the wall 3m away, for 2 or 3 minutes (120/180s) at a time (Fig. S5). They were instructed to remain motionless and keep their eyes open, blinking if they needed to.

2. *Finger movement*

Subjects were cued with a word displayed on a bedside monitor to move fingers independently during 2-second movement trials. They typically moved each finger 3-5 times during each trial, but some trials included many more movements. A 2-second rest trial (blank screen) followed each movement trial. There were 30 movement cues for each finger, and trial types were interleaved randomly (typically 100-150 totally movements per finger). Finger position was recorded using a 5 degree-of-freedom dataglove (Fig. 2). Event markers were calculated marking the initiation of movement following a cue, and the peak of each finger movement . "Rest" events were defined during random periods occurring at least 500 ms from any movement initiation or termination, and separated by at least 250 ms from any other rest event (there were typically 150-250 such rest events). See figure S20 (A) and (B).

D. Spectral Calculation

Figure S6 illustrates the steps taken to transform the raw voltage time series from each electrode into power spectra. The data was re-referenced in terms of neigh-

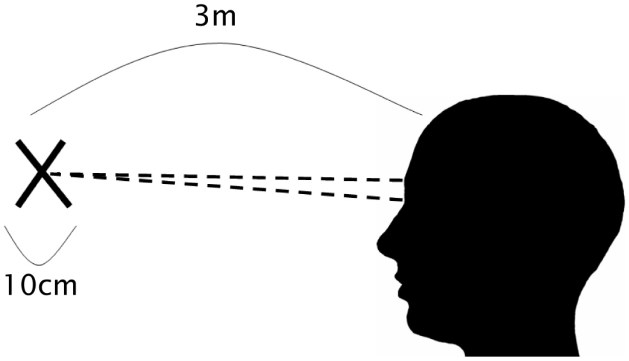

Figure S5: Subjects fixated on a 10cm cross 3 meters away for 2-3 minutes during the fixation task.

boring differential pair channels, $V(t) = V_i(t) - V_j(t)$ (our 32 electrode arrays have 52 differential pair channels each, as in figure S4). This significantly reduced the overall correlated noise in the signal. This reduction is to be expected, since it removes various non-local contributions to the signal, while the signal of interest originates from the neurons (about 5×10^5 of them) immediately underneath each electrode. Note that a common average re-reference, for example, increases the uncorrelated noise (noise-floor) by a factor of $\sqrt{Numberelectrodes}$, and is therefore not advisable. Next, the time series were broken-up into 1 second long intervals, overlapping by 0.5 second (we did not examine phenomena with frequencies below 1Hz). Each of these epochs was windowed

$$V'(\tau, m) = V(\tau + \tfrac{1}{2}mT)H(\tau) \qquad (1)$$

with a Hann-window $H(\tau)$ of the form

$$H(\tau) = \tfrac{1}{2}\left[1 + \cos\left(\frac{2\pi}{T}\tau\right)\right] \qquad (2)$$

inside time interval$-T/2 \leq \tau \leq T/2$ with $T = 1$ sec, and $H(\tau) = 0$ at all other times (see figure S7). The power spectral density (PSD) for each epoch follows from the Fourier transform

$$P(f, m) = \frac{1}{T}\left(\sum_{\tau=1}^{T} V'(\tau, m)\, e^{i2\pi f\tau}\right)^2 \qquad (3)$$

Each of these individual spectra is quite noisy; examples of this are shown in Fig.6.E. Taking the average PSD over all epochs

$$P_R(f) = \frac{1}{M}\sum_{m=1}^{M} P(f, m) \qquad (4)$$

quiets this down into a smooth PSD for each channel pair, as illustrated by the green line in Fig.2 of the main text and again in Fig.6.F. (The same curve but shown with a linear frequency scale instead of a logarithmic one.)

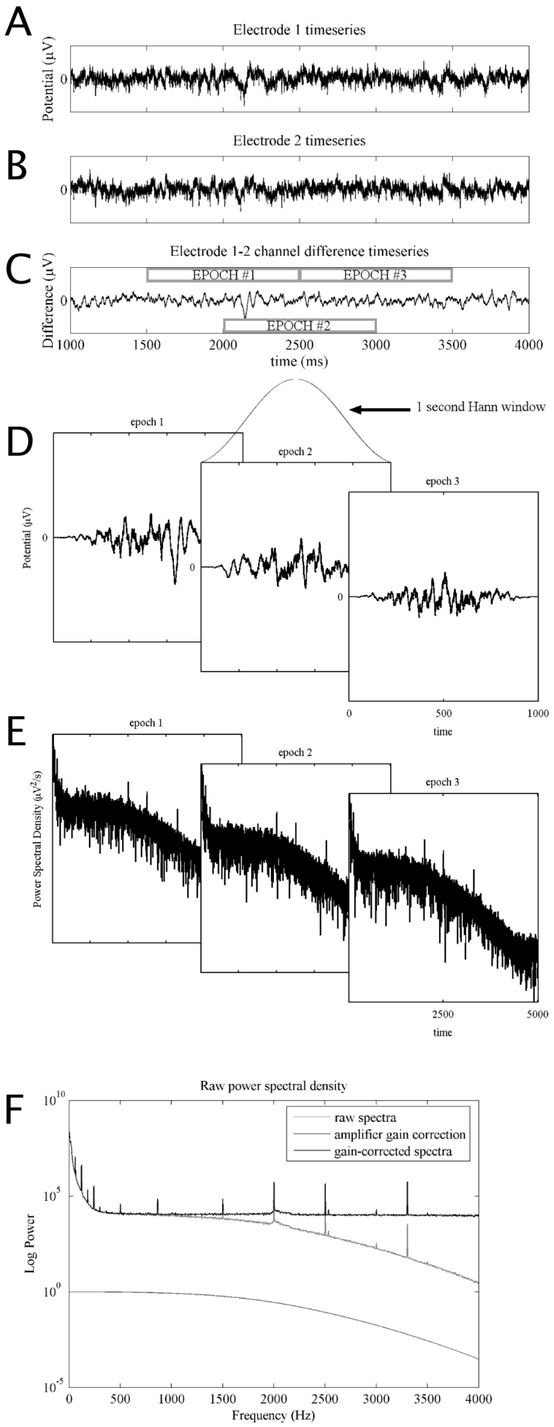

Figure S6: Power Spectral Density Calculation: (A) and (B) show voltage time series of 2 nearby electrodes during rest. Their difference, shown in (C) (local pair electrode referencing) is boken-up into 1 second long segments, and each is Hann windowed (D) and Fourier transformed to generate the power spectra, shown in (E). Each is very noisy, but their average in (F) is smooth.

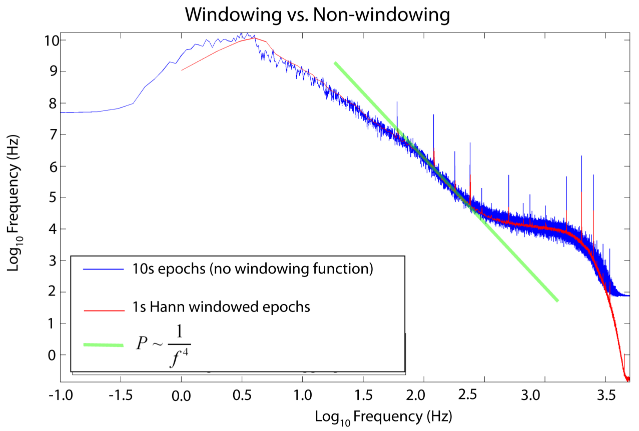

Figure S7: Using a Hann-window does not introduce an overall effect on the shape of the PSD.

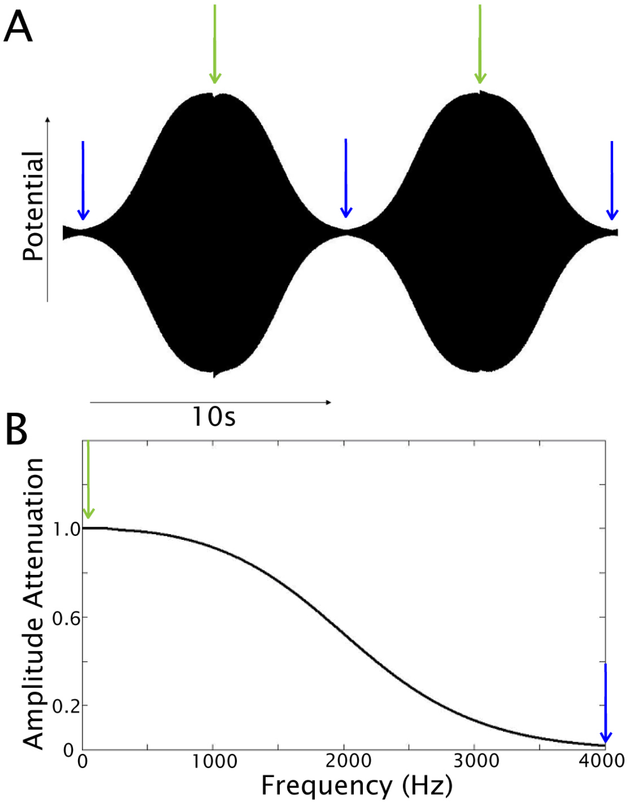

Figure S8: The amplifier roll-off was calculated by repeatedly scanning through frequencies at a fixed from 15Hz (green arrows) and 4000Hz (blue arrows), and the amplitude attenuation as a function of frequency could be determined directly from the timeseries. **(B)** Illustrates this measured frequency-dependent amplifier amplitude attenuation, $R(f)$, at 10KHz sampling rate.

The combination of the 50% window overlap and Hann window insured that each timepoint contributed equally to the PSD.

Each PSD represents the Fourier transform of the voltage auto-correlation function

$$G(\tau) = \int dt\ V(t+\tau)V(t) \tag{5}$$

averaged over the entire time interval of the experiment. Because of this, phenomena like the power-law we find below have directly interpretable implications for the correlation of the voltage timeseries.

E. Amplifier Characterization and correction of spectra

The properties of the amplifiers proved a major issue in the data analysis because of their (surprisingly high) noise floors and frequency-dependent amplitude attenuation (roll-off filter). The use of higher quality amplifiers was impossible because of the requirement for FDA (Food and Drug Administration of the USA) approved instrumentation with human subjects. These recording amplifiers had built in roll-off filters which were not removable, and had a set low-pass filter with maximum value for each sampling rate. Because of the proprietary nature of the hardware/software, and the clinical prohibition from modification of these, it was not possible to avoid the low-pass setting. Therefore, we had to experimentally characterize this low-pass filtering, and correct our analyses accordingly.

We determined the amplitude attenuation function $R(f)$ (roll-off factor) independently by means of an external function generator by repeatedly sweeping (at a rate of 10 seconds) at fixed amplitude through all frequencies between 15Hz and 4000Hz at the 10KHz sampling rate setting, and between 10 Hz and 300 Hz at the 1KHz sampling rate setting. This $R(f)$ followed a Lorentzian type shape, as expected, and is shown in figure S8. We divided our PSD's by this filter, $P(f) = P_R(f)/R(f)$. It completely removed the roll-off from the PSD's, as illustrated in figure 1 of the main text and also in figure S6F. After correction for this roll-off, a definite noise floor came into focus, revealed by the asymptote in figure S6F.

This noise floor is atypically large for experimental amplifiers, but we empirically established that they are the source. This noise resides in the amplifiers, and does not reside inside the brain. The amplifier noise floor was determined experimentally by measuring the potential across an equivalent conformation of resistors, shown in figure S9. This was done in situ in the sense that parallel clinical amplifiers remained attached in parallel during the recording. The empiric values of the noise floor were determined by calculating spectra of these resistor measurements using the identical method used with brain signals, and detailed above. Following on-line measured impedance with electrodes on the brain surface, $R2$ was set to 10kΩ and $R1$ to 0.5Ω (although noise values were robust against increasing $R1$ to 1k Ohm). The result is shown in figure 1 of the main text.

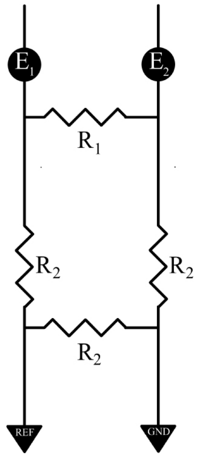

Figure S9: Equivalent resistor arrangement during amplifier noise floor estimation. Following on-line measured impedance, $R2$ was set to 10kΩ and $R1$ to 0.5Ω (although noise values were robust against increasing $R1$ to 1kΩ).

We do not present further details of our independent amplifier noise floor experiments, because the cortex data suggests that the floors vary slightly between subjects, electrodes, and recording days. Therefore we treated the noise floor as a free (to be fitted) parameter in the data analysis. The distributions of measured noise floors from this resistor arrangement, and fit noise floors (described below) were overlapping (consistent with one another), and are shown in figure S17.

F. Fitting the PSD at High Frequency

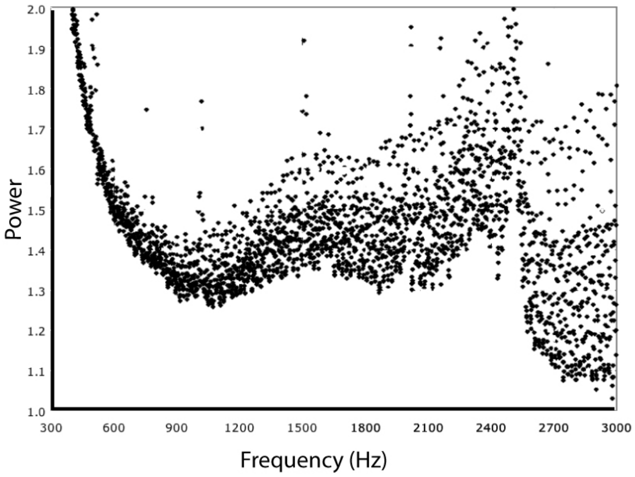

Figure S10: PSD of a single pairwise re-referenced signal in subject 1, after removal of amplifier roll-off.

We fit our experimental high frequency power spectral density to the form $P \simeq Af^{-\chi} + C(f)$. The first term, $Af^{-\chi}$ is the power law shaped power spectrum we wish to explore, and $C(f)$ is the amplifier noise floor. If $C(f)$ is known exactly, then the value of the exponent, χ is straightforward, and given by the slope of the data on a plot of $ln\left(P(f)\right)$ vs. $ln\left(f\right)$ after $C(f)$ has been subtracted.

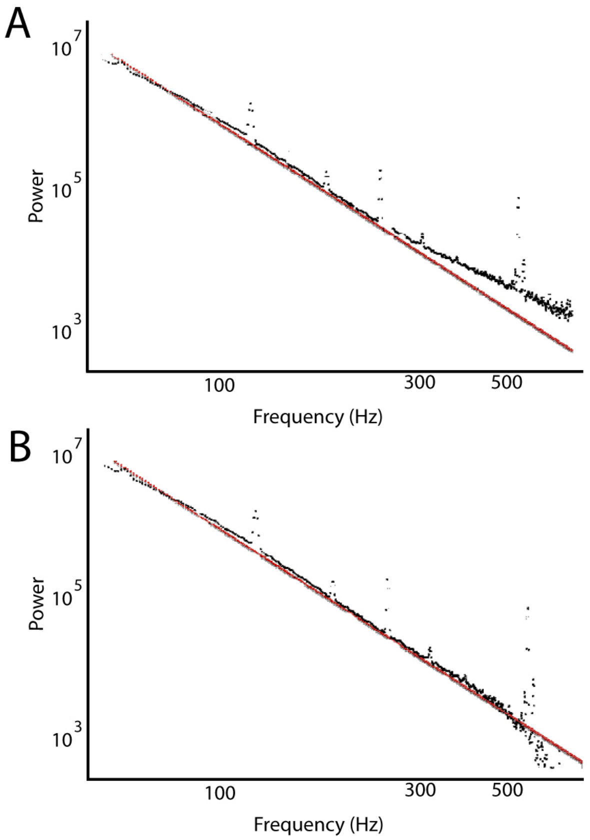

Figure S11: Power law fit of S1 in frequency range $80 < f < 200$ for (A) noise floor $C = 15500$ and (B) $C = 12500$ (amplifier units), demonstrating that the exponent $\chi = 4$ is insensitive to the noise floor value at these lower frequencies and that (B) provides a better global fit (arbitrary units of power).

While it may have some frequency dependence, we chose to approximate $C(f)$ as a constant, C, because we could not identify a robust frequency-dependent trend across spectra, and, in our noise measurement across a series of resistors, $C(f)$ was roughly linear in the 80-600Hz range we fit our data in (Fig. S12). One choice for how to estimate C would be the asymptote after roll-off correction, but this does not work because of an unknown contribution that is non-uniform, and begins at $\sim$ 1kHz (Fig. S12 and S10). The origins of this high frequency contribution remain unresolved: It might reflect sensitivity to details of the amplifier roll-off function $R(f)$ (amplified by the division), it might originate from the internal electronics of the amplifiers, or tt could reflect an

external high frequency source that is not fully removed by the nearest neighbor pair referencing.

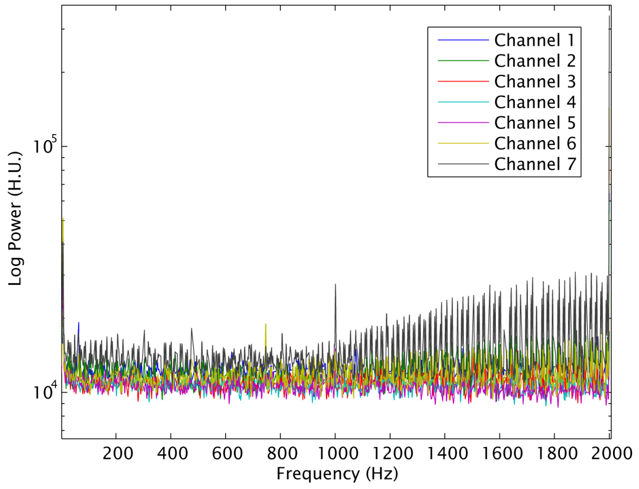

Figure S12: Measured spectra across the equivalent resistors, following the recording for subject 2. The first 7 electrode-pair channels are shown, after correcting for the amplifier frequency response (roll-off). 60Hz harmonics omitted. Note that all are approximately linear (with slope zero) in the fitting range, and that some have an anomalous contribution in the range above $f > 1000Hz$ (arbitrary units of power).

In any case, a direct measurement of these noise floors offline in our fitting range (Figs. S12 and S17) produces a characteristic range of values, and we found that the measured electrode-pair channel noise floors varied across electrodes, within a distribution, and also on a day-to-day basis. They may also have some sensitivity to the total power in the signal, as shown in Fig. S16A (with "fit noise floor" described below). In light of these uncertainties, we felt that the only proper approach is a self-consistent fitting procedure limited to the frequency range, $\simeq$80 Hz $< f <\simeq$500 Hz.

Before detailing the self-consistent noise floor subtraction, observe that figure S11 demonstrates that the power law with exponent $\chi \simeq 4$ is stable and insensitive to the exact value of the noise floor in the frequency range $80 < f < 200$ Hz. Note that the noise floor choice $C = 15500$ (amplifier units) extends the power law fit all the way to 500 Hz.

We fit the PSD to the form $P(f) \simeq Af^{-\chi} + C$ in the frequency interval 80 Hz $< f <$500 Hz. The simplest approach might be to plot $\log(P(f) - C)$ versus $\log(f)$ for a range of guesses of the noise floor C, and choose the value of C for which the curve straightens out best into a straight line, and then letting the slope of that line being the value of the exponent χ.

However, an infamous mistake in this procedure is to apply global least squares fit, and leave it at that. On a log-log plot, that assigns too much weight to the highest density of datapoints, at high frequency, where the low power and high relative influence of the noise floor

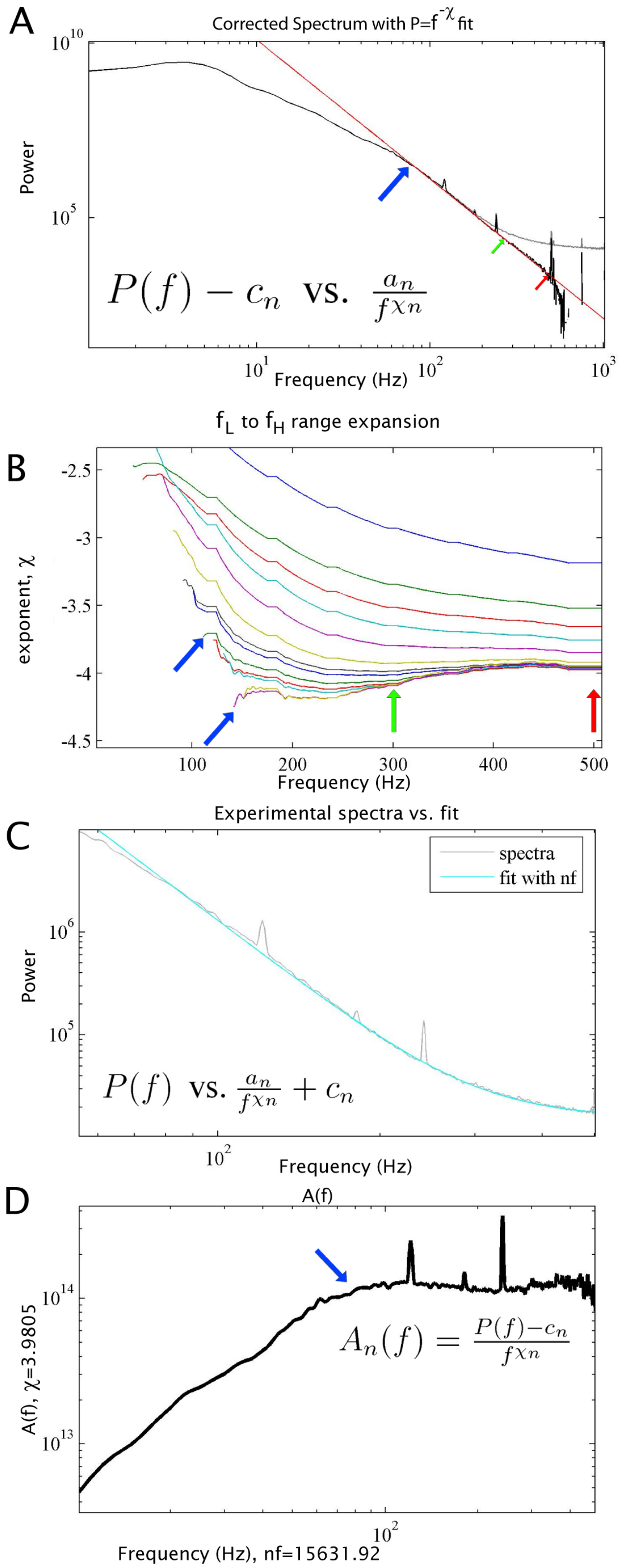

Figure S13: Illustrations of the recursive range shrinking fitting protocol of the PSD to the form form $P(f) \simeq Af^{-\chi}+C$. *(See text for details)*

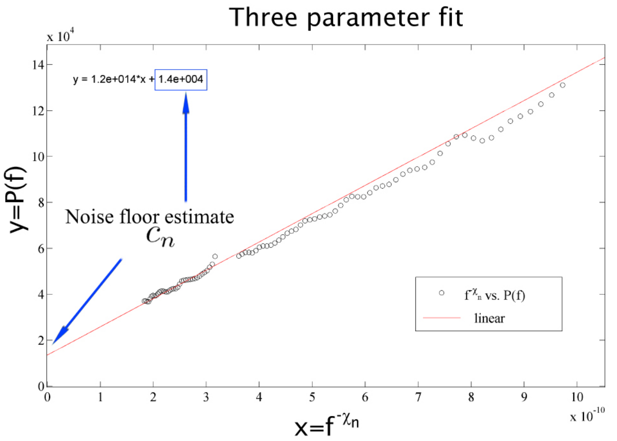

Figure S14: Illustrationof the 3-parameter fit protocol to recursively estimate the appropriate noise floor. f^{χ_n} is plotted vs $P(f)$, and the new value of the noise floor, c_n is estimated by the intercept with the y axis at $x = 0$.

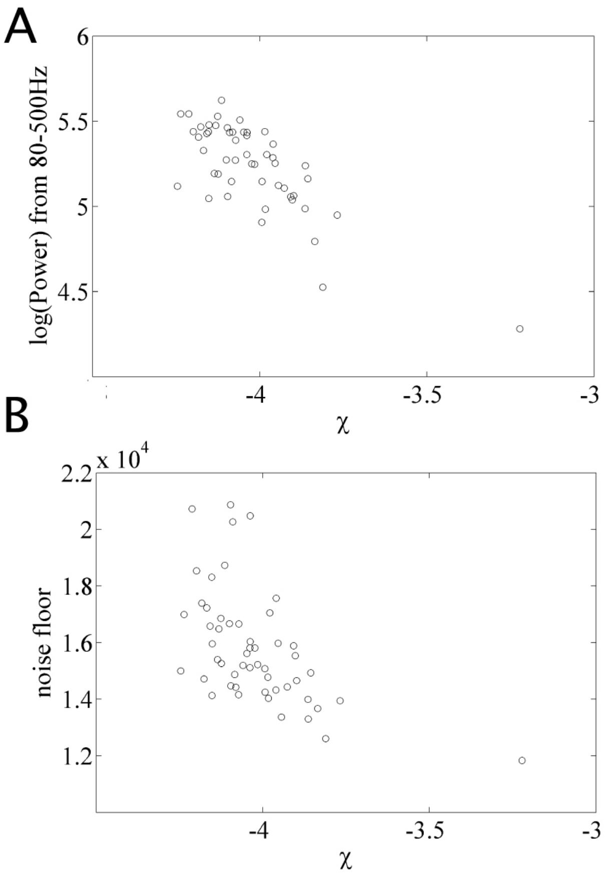

Figure S15: **(A)**Correlations between the fitted values of the exponent χ and the total power (amplifier units) in the signal between 80 Hz$\leq f \leq$ 500 Hz in the individual electrode pairs of subjects S1 and S2. Red dots indicate channel pairs rejected because of vasculature as discussed in the text. **(B)** Correlations between the fitted values of the exponent χ and the fitted noise floor C (amplifier units) in individual electrode pairs of subjects S1 and S2.

make the data noisiest. In reality, a fit should be stable throughout the fitting range, and we employed a technique which is robust against range shrinking to a subrange within the total fitting range (illustrated in Fig. S13).

The PSD crosses over to a different form at the low end, near $f_0 \simeq 75$ Hz. The noise floor becomes the important factor at the high end, $f > 400$ Hz. The conventional approach to this, which we employ, is to determine local slopes $\chi(f)$ by performing least square fits to the curve over only narrow frequency intervals, from a low cutoff frequency, "f_L" to a high cutoff frequency, "f_H" - $f_L < f < f_H$ along the curve, and plot these $\chi(f)$ estimates for a range of choices of the noise floor C. 1. If the value of the locally fit exponent varies greatly for different values of f_L and f_H within the global fitting range, then the PSD is not well explained by a power-law in that global range. The best fit for C is the $\chi(f)$ curve with the widest flat plateau. The best value for χ is that plateau value. A potential problem to be avoided with this range-shrinking approach is that local slopes become increasingly noisy for narrow fitting intervals $f_L < f < f_H$, and the plateaus can then drown in the noise. In light of this, and because of the need to automatize the fitting procedure due to the large amount of data, we use the following recursive protocol.

It starts with an initial guess for value of the exponent, χ. That value is used for an initial estimate for the noise floor C by means of a straight line fit of the form $P(f) = Ay + C$ as function of $y = f^{-\chi}$, using the high end of our frequency range, 250 Hz$\leq f \leq$ 490 Hz (shown in figure S14). Next, we use this estimate of C to improve on the estimate of χ by performing least square fits to $\log(P(f) - C)$ versus $\log(f)$ between $f_L < f < f_H$ with the $f_L < f_H$ interval covering (expanding and shrinking over) the entire frequency range 80 Hz $< f <$500 Hz. We plotted these estimates $\chi(f_L, f_H)$, as illustrated in Fig.13.B, with f_H along the horizontal axis and different lines representing different values of f_L. (The onset of the lines mark the values of $f_L + 20$Hz, because f_L is always smaller than f_H. Different values of f_L are denoted by different color lines.) The quality of each fit is reflected by the presence of horizontal segments in the curves, by the width of those segments with f_H, and by collapse of those segments with the curves at nearby f_L. For example, from figure S13B, we would conclude that $\chi = 3.95 \pm 0.1$; and then use this estimate to start the next iteration cycle by setting $\chi = 3.95$ in $y = f^{-\chi}$ for the next $P(f) = Ay + C$ estimate of the noise floor C; and so on. This iteration scheme always converged for our data. The quality of the fit can be further judged by plotting $A(f) = (P(f) - C)f^{\chi}$, to check how well the final amplitude A is truly a frequency independent scalar (in the frequency range of interest), see figure S13D.

The curves $\chi(f_L, f_H)$ in figure S13 B have distinct

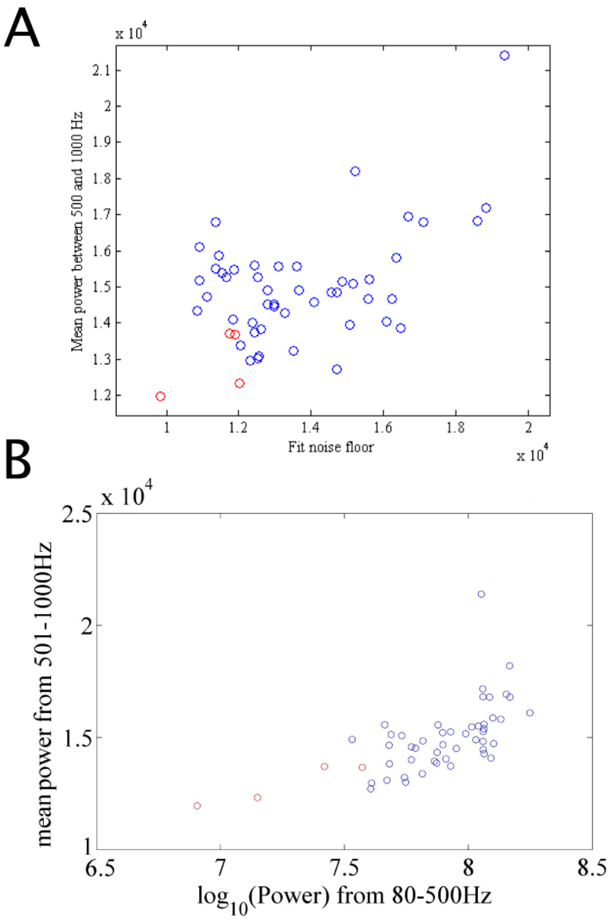

Figure S16: **(A)** Correlations between the fitted noise floor C and the average power in the high frequency range 500 Hz$\leq f \leq$ 1000 Hz, for individual electrode pairs of subjects S2. **(B)** Correlations between the average power in the high frequency range 500 Hz$\leq f \leq$ 1000 Hz and the total power between 80 Hz$\leq f \leq$ 500 Hz, for the individual electrode pairs of subjects S1 and S2.

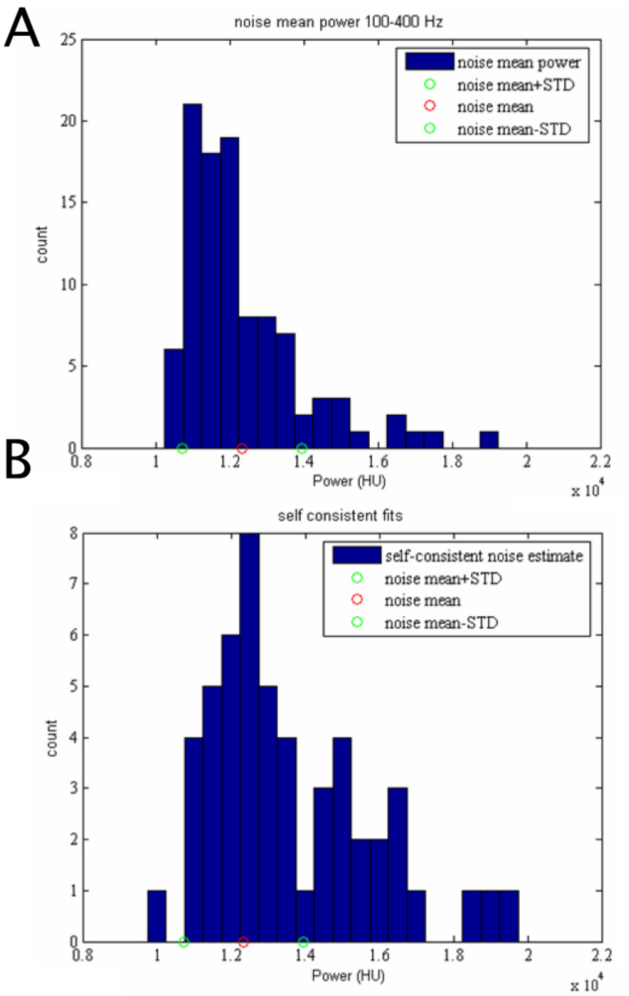

Figure S17: This shows the difference in the distribution across channels for the experimentally found calibration noise floor (A) (averaged between 100-400 Hz), and the self-consistent fits (B) for S2. The red and green dots in both panels indicate the mean +/- STD of the experimentally found calibration noise floor. The calibration was performed with an identical set of clinical amplifiers in parallel, immediately following the experimental recording (although in a different room). Note that the channel distribution for the self-consistent fit from experimental data has a slight rightward shift, indicating that there may have been additional contributions to the noise floor in the subject's hospital room that couldnt be accounted for in the calibration.

minima for intermediate values of f_L and f_H. This shows how easily the exponent χ can be underestimated. Choosing f_L too close to $f_0 \simeq 75$ Hz, where the PSD crosses over to a less steep curve, systematically underestimates the exponent. Under estimating the noise floor C has the same effect at the high frequency side; and results in an up-swing in the $\chi(f_L, f_H)$ curves at high frequencies. Overestimating the noise floor leads to negative values of $P(f) - C$ at high frequencies and a collapse to erratic behavior in the $\chi(f_L, f_H)$ curves at high frequencies. It is significant therefore that the curves deepen during the iteration process in the interval where f_L and f_H both take intermediate frequency values.

We performed this fitting protocol on the electrode pair averaged PSD of subjects 1 to 4, with result $\chi = 4.0 \pm 0.1$. The upper limit of the fit was determined by the frequency at which the spectra ran into the noise floor, but all were between 500 and 600 Hz (figure 2 of the main text). Note that the electrode arrays in those subjects are located at different parts of the motor cortex. We also applied the same fits also to the more noisy individual electrode pair PSD's from all four subjects, leading to a narrow distribution of exponents χ centered at the same value $\chi = 4.0 \pm 0.1$ and with a width consistent with the above error estimate. This is also shown in figure 4 of the main text and addressed there.

A logical follow-up issue is to check for possible systematic effects in the individual electrode pair signals. The total power in each electrode varies greatly, by about 10 percent, as seen in Fig.S15. This has various reasons. The most important one is probably the proximity of blood vessels and/or variations in quality of electrode-pia-cortex contact. The most anomalous (weakest) signals were cleanly correlated visually, in figure S4, with electrodes sitting on vasculature and were removed from consideration in further levels of analysis.

Figure S15A tests for correlations between the value of the fitted exponent χ and the total power in each channel pair. A weaker signal makes that the PSD drowns into the noise floor at a lower frequency. That is likely to result in a poorer fit and to a systematically smaller value of χ, but figure S15B shows that this effect is weak.

Figure S15B tests for possible systematic correlations between the fitted values of χ and C. It appears that such correlations are weak compared to the channel pair statistical noise. The fitted values of the amplifier noise floors C in specific electrode pairs vary widely, by roughly 20 %. This is not due to the fitting protocol, because in figure S16A the fitted noise floors correlate well to the average high frequency power (at 500 Hz $\leq f \leq$ 1000 Hz).

Finally, figure S16B shows that this average 500 Hz$\leq f \leq$ 1000 Hz high freq power is slightly correlated to the total power between 80 Hz $\leq f \leq$ 500 Hz, possibly suggesting that the amplifier noise floors vary with input power.

In conclusion, figures 15 and 16 suggest some systematic effects, but they are rather weak compared to statistical variations between the electrode pairs. It does not seem proper to pursue them further at this point.

G. Adjustment for a more complex form

Examination of figure 2 of the main text shows a clear power-law in the cortical spectrum above 80Hz, in all 4 subjects sampled at 10kHz. The PSD changes its slope below visually $f_0 \simeq 75$ Hz. This knee at f_0 is less pronounced in subjects 2 and 3 because there are prominant $\alpha - \beta$ peaks.

These rhythms obscure possible underlying broad band features. We employed two approaches to get around this and assessed the underlying PSD at frequencies below f_0. Both involved data from experiments that we performed at a lower, 1kHz, sampling rate:

(1) The first is that we can take advantage of spatial variation in these low frequency rhythms of the ECoG recording. While most of the electrode-pair channels that we record have the α and/or β rhythms, not all do. We examined the PSDs of 16 subjects (subjects 5-20, see the table in figure S1) performing the same fixation task. We were able to visually identify 91 channels in which the PSD did not show α and β rhythms. Selection bias was avoided by visually examining spectra on linear axes (see figure S18). The lower frequencies of these selected channels could then be examined for scale-free, power-law properties, as they were devoid of the peaked rhythms.

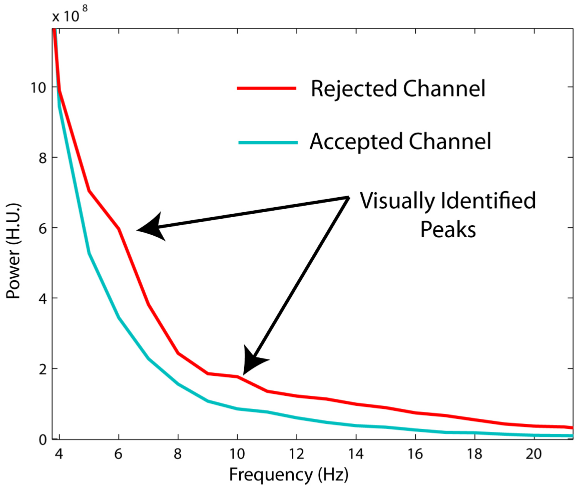

Figure S18: Electrode pair channels with $\theta/\alpha/\beta$ were visually rejected by viewing on linear axes, so that selection bias for "power law" shape - linear on log-log axes could be avoided.

(2) One goal of this examination of scale-free (power-law) properties in motor cortex was to see how the power spectrum changes when the brain becomes active. Our experience with cortical change during motor movement made that a natural setting to examine activity, but the α and β rhythms are ubiquitous in motor areas. The second strategy was to use variation in the spectra to decouple and remove the rhythms from the broad band features. We developed the technique to this in a different study using a naive, principle component-type, analysis (reproduced below) and we obtained qualitative consistency with the results of fixation data. This was ultimately feasible because a finger movement task that we used resulted in spectral change in which the low frequency rhythms varied differently from a broad-band spectral change from 5-200 Hz. It was important to examine this movement task, because it allowed us to look at how the measured power-law phenomena (demonstrated below) behaved when the brain became active, in the absence of the α and β rhythms.

The 1 kHz sampled data (subjects 5-21) were limited by sampling rate and by built-in proprietary constraints of FDA approved amplifiers to spectra which ended at a high value of f = 200Hz. In order to characterize the power law at $f >$ 80Hz, this was insufficient, and we had to examine data recorded at a higher sampling rate. However, armed with the high quality fit of $P \simeq \frac{1}{f^4}$ above 80Hz, we could examine the lower frequencies, and impose the contstraint that any formalized structure in the PSD must fit $P \simeq \frac{1}{f^4}$ for large f.

a. naive fit The first step in examining whether there is evidence for a power-law of some kind in the PSD is a naive fit of $P \simeq \frac{1}{f^\chi}$ within a low frequency range, for

the 1kHz data. To do this, spectra were calculated in the same way as for the 10kHz data. They were then corrected for frequency-dependent amplifier attenuation (roll-off). They were not corrected for noise floor, because the power of the signal in the fitting range is many orders of magnitude larger than the noise floor contribution. The naive power law fit to this averaged PSD over 15 Hz $\leq f \leq$ 80 Hz yielded the value $\chi_L = 2.46 \pm 0.32$ (see figure 3 of the main text and figure S19).

b. Lorentzian adjustment Rather than the naive fit, we propose also the following (naively chosen) phenomenological global fitting form

$$P_A(f) \simeq \frac{A\ f^{-\chi_L}}{1+\left(\frac{f}{f_0}\right)^{\chi_H}} \qquad (6)$$

and apply it over the frequency range 15 Hz $\leq f \leq$195 Hz for the 91 channels of 1KHz data which did not have blatant peaks at lower frequencies, nor inordinately

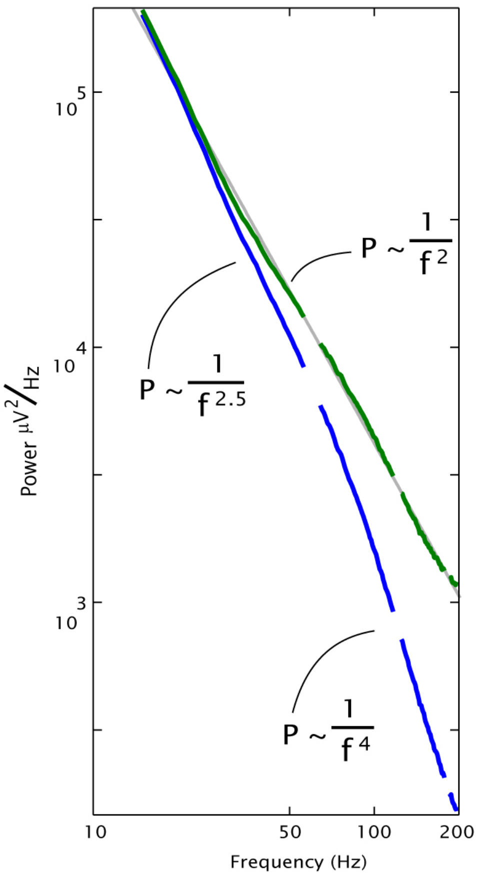

Figure S19: Low frequency fits to the 91 channel pair PSD's from 16 subjects, sampled at 1 kHz over frequency range 15 Hz $\leq f \leq$ 80 Hz (blue), and over 15 Hz $\leq f \leq$ 195 Hz after multiplying the PSD's with $1+\left(\frac{1}{75}\right)^2$ (green). The lines are the averaged spectra from all electrode-pair channels in all subjects. Note that this transformation pulls a straight line with the form $P \sim 1/f^2$ out, across the entire fitting range.

large noise floors at higher frequency ranges. This is an approximation of a two-Lorentzian form, where the 2nd factor, $f^{-\chi_L}$, is obtained by $\frac{1}{1+\left(\frac{f}{f_L}\right)^{\chi_L}} \longrightarrow f^{-\chi_L}$ for $f \gg f_L$ and is therefore valid in the case that the lower "knee" at is well below 15Hz. We imposed the condition that the two exponents add-up to the value $\chi = \chi_L + \chi_H = 4.0 \pm 0.1$ (established above, for data sampled at 10kHz). For each electrode-pair channel's PSD, $P(f)$, we iteratively updated estimates of χ_L and f_0, until they converged on stable values, by:

(1) Calculating an updated χ_L as the slope of the least-squares linear fit of log(f) vs. $\log\left(P(f)\left(1+\frac{f}{f_0}^{4-\chi_L}\right)\right)$ on the fitting range 15 Hz $\leq f \leq$195 Hz.

(2) Calculating an updated f_0 as the value of f_0 in the fitting range 15 Hz $\leq f0 \leq$195 Hz for which the slope of log(f) vs. $\log\left(P(f)\frac{\left(1+\frac{f}{f_0}^{4-\chi_L}\right)}{f^{\chi_L}}\right)$ was closest to zero.

This produced χ_L=2.01±0.18 and f_0=77Hz±14Hz, across the 91 electrode channel pairs in subjects 5-20.

H. Removal of the α and β peaks

While 1kHz data spectra could be fit to low frequencies in channels which lacked the low frequency α/β rhythms, we wanted to know how the PSD changed with increased neural activity. Based upon previous studies, we knew that the cortical PSD in motor areas changed reliably during motor movement, reflecting an increase in activity of the local neuronal population. In order to examine just the scale-free, power-law activity however, we needed to employ a technique to remove the α and β rhythms, since they are ubiquitous in motor areas.

Using a technique developed and elaborated in the manuscript *Decoupling the Cortical Power Spectrum Reveals Real-time Representation of Individual Finger Movements in Humans* by K.J. Miller et. al. in J Neurosci, 2009; **29**, 3132, we were able to decouple and remove the α and β rhythms from spectral samples during periods of finger movement and rest, in sites that showed movement-associated cortical change in the 76-100Hz range (as detailed in *Spectral changes in cortical surface potentials during motor movement* by K.J. Miller et. al. in J Neurosci, 2007;**27**, 2424). In abbreviated form, the decoupling method is as follows. Subjects are given visual cues to perform repeated finger movement (Fig. S20 A). Individual fingers were moved several times in response to each visual cue, and position was recorded using a dataglove (Fig. S20 B). The peak displacement of each finger movement was marked with an event marker, τ_q (e.g. black arrow). Event markers, to characterize non-movement spectra for the "rest state" were chosen at random times at least one-half sec-

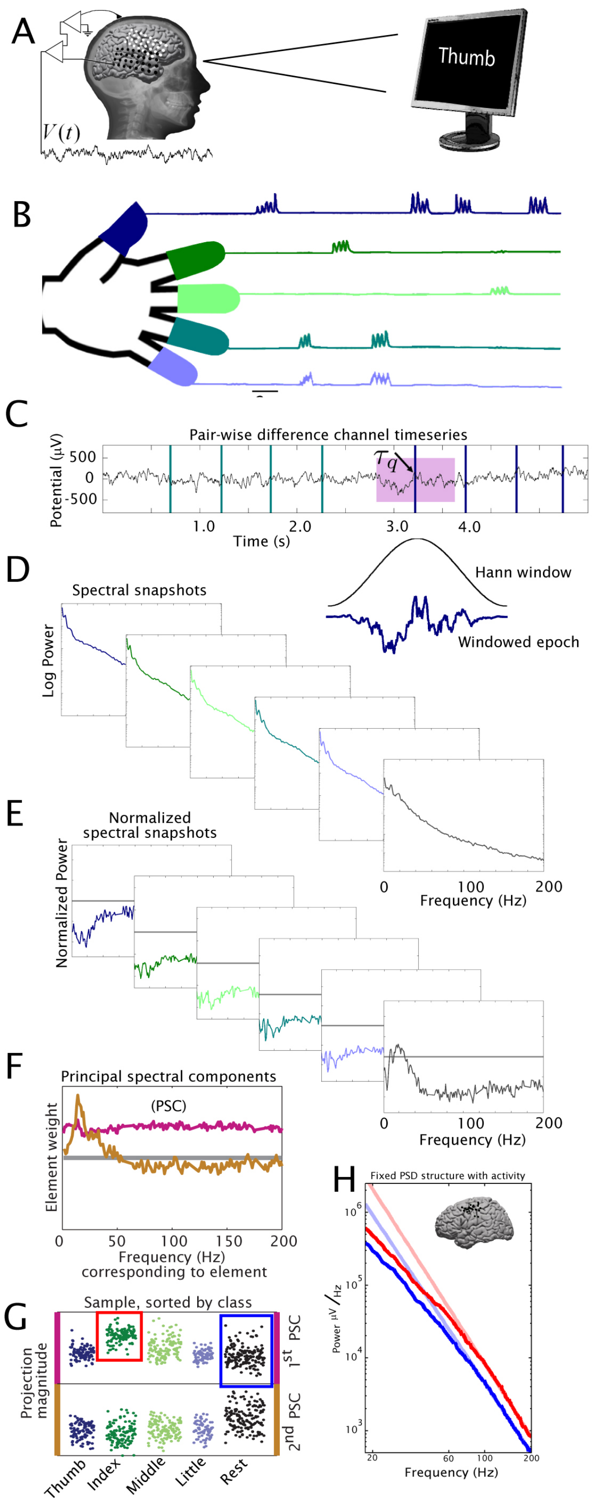

Figure S20: Using variation in spectra during finger movement, the so-called α and β peaks in the PSD can be extracted. *(See text for details)*

ond from any movement event, and one-quarter second from each other. A pair-wise electrode difference channel, $V_n(t)$, is shown with these event markers (colored) in figure S20 C. A 1 sec Hann widow $H(t)$centered at each event marker, is imposed on the data to select the epoch corresponding to that specific event marker. Samples of the power spectral density (PSD, $P_n(f, \tau_q)$) associated with each event marker are obtained for each epoch by Fourier transformation from these epochs (Fig. S20 D). Each individual epoch power spectrum is normalized by dividing through by the mean power at each frequency, and taking the log (Fig. S20 E), and a Principal Component decomposition method is applied to these normalized spectra. Principal Spectral Components (PSCs) are calculated across these across these sets of epoch power spectra (Fig. S20 F). The first is primarily flat across all frequencies (pink), and the second is peaked in the classic α/β range (brown). Figure (S20 G) shows back projections of the first PSC (upper - pink) and second PSC (lower - brown) to the power spectral density samples, sorted by class. Note that the first PSC is specific for forefinger, and the second shows significant decrease for all movement classes with respect to rest (consistent with ERD). We can then reconstruct average spectra from one finger movement class (in this case, forefinger), and rest without the α and β rhythms, which are captured by the 2nd and 3rd components. The average of these reconstructed spectra, without the 2nd and 3rd components, is shown in figure 4 of the main text, and is reproduced the last panel of figure S20.

After this decomposition, we reconstructed the mean spectrum for each channel during rest, $P_R(f)$, and during movement, $P_M(f)$. Each of these were approximately linear on a log-log plot after multiplication by $\left(1 + \frac{f}{75}^2\right)$, and had slope consistent with $\chi_L = -2$ (used to produce figure 4 of the main text, but not to quantitatively test the shift). In order to assess whether there was a shift in exponent during movement, we examined $\log(f)$ vs $\log(P_M(f)/P_R(f))$, and found that the slope of this quantity on the interval 25-195Hz was essentially zero -03 $\pm$ .09, ($\pm$SD, N=25, p=0.104, by paired t-test). This implied that there was no shift in exponent, χ_L with movement (when local neuronal populations became active). Because there was no shift in exponent, the average values of $(P_M(f)/P_R(f))$ determine the change in the coefficient, A, of the power law process (geometric mean $R = 1.76$, with a variation (standard deviation) of order 0.31 (maximum 2.47, minimum 1.29, N=25, $p = 5.9{*}10^{15}$ by t-test of log-ratio vs. 0)). Please note that this number reflects a reasonable range, rather than a fundamental quantity.

II. SIMULATING THE ORIGIN OF THE ECOG POWER SPECTRUM

Each ECoG electrode lies sub-durally on the cortex (figure 5A of the main text), with $\simeq$5mm^2 of platinum surface area exposed. The cortical volume immediately beneath this exposed area, contains approximately 10^5 neurons, and a typical neurons has $\simeq 10^4$ synaptic inputs.

The electric voltages observed by the ECoG electrodes probe the same features as EEG and MEG but with superior spatial and temporal resolution. Following the conventional EEG/MEG literature (*See the Nunez, et. al., and Hamalainen, et. al. references of the main text*), the cortex is interpreted as an ionic liquid filled with current sources $\vec{J_s}$. These current sources arise as currents flowing in and out of the neurons. The voltage at a specific electrode at position is then the superposition of all current sources i:

$$V(t) = \sum_i \frac{\nabla \cdot \vec{J_s}(i)}{4\pi\sigma d_i} \qquad (7)$$

with $\vec{\nabla} \cdot \vec{J_s}$ the divergence of the current of source i, σ the electric conductivity of the ionic liquid, and d_i the distance between the current source and the electrode. (This assumes a uniform medium approximation and some other simplifications.) It is custom in the EEG/MEG literature to sort the current sources into pairs, i.e., to think in terms of current dipoles, to combine them at various levels of coarse graining into effective meso-copic dipoles, and to treat those as instantaneous.

For example, each synaptic event induces a post-synaptic current influx. The amplitude and sign of this input current varies between synapses. The post synaptic charge builds up due to current influx, with a timescale of approximately $\tau_1 = 3$ msec. This charge spreads along the dendrite, overall diffusing toward the soma, and, as it does so, it also ohmically leaks back to the ionic extracellular bath (accordingly leaky cable equation) over a much longer time scale. The ECoG temporal and spatial resolutions are such that (in the passive dendrite approximation) the in and out flux components of every dipole are separable in time, and also that the temporal correlations between individual dipoles become visible. They are at the origin of the ECoG broad band.

The dominant contributors to the cortical change are believed to be the synaptic current events of the cortical pyramidal neurons, which have a primary dendrite that lies roughly normal to the cortical surface, so that the ensemble of synaptic current dipoles have a common directionality. Hamalainen, et. al., in their 1993 paper *Magnetoencephalographytheory, instrumentation, and applications to noninvasive studies of the working human brain.*, estimated the isolated, individual transient dipole moments, from each synapse to be of order $|\vec{q}| \approx 20$ fA$\cdot$ m. Murakami and Okada, in their 2006 paper *Reconstructed shapes, firing patterns and intracellular current dipole moments of layer V neocortical pyramidal cells*, found that the net dipole moment produced by an entire neuron, in response to various kinds of more global input is of order $|\vec{q}_{global}| \approx$1 pA$\cdot$ m, in agreement with the Hamalainen estimate, since only a subset of synapses are activated during a common input.

A. Simulation

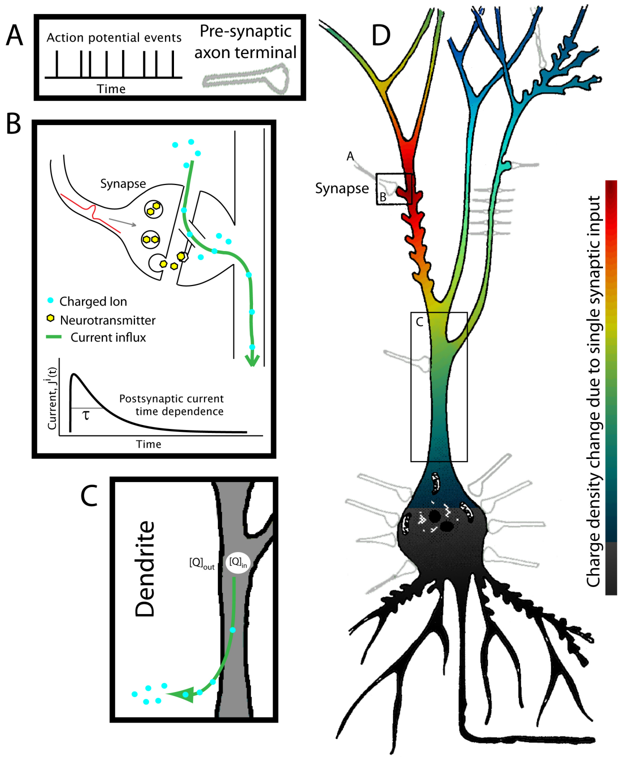

Figure S21: In the simulation, a randomly generated set of delta-function action potentials arrival times **(A)** is convolved with a characterized, exponentially decaying, post-synaptic current **(B)**. The time-dependence of the dipole is approximated by the dendritic trans-membrane current, which is proportional to the difference in transmembrane charge density **(C)**. These events are all concurrent within the neurons beneath one of our electrodes **(D)**. (Note that the spatial relationship, and many other factors are not incorporated into this simple model). The neuronal outline was inspired by Hamalainen, et. al., in their 2001 paper: *Magnetoencephalographytheory, instrumentation, and applications to noninvasive studies of the working human brain.*.

.

While there are many potential models that are mathematically consistent with the form of the spectrum that we found experimentally, we performed a simplified simulation of only one such model. We simulated the time-course of current dipole sources in the dendritic structures of the neurons beneath each of our electrodes with three simple processes. Each pyramidal neuron, beneath each one of our electrodes, receives 6000-25000

synaptic inputs, and we model one with 6000 synapses. The series of input action potential arrival times from each pre-synaptic neuron was modeled with a Poisson-distribution in time. When an action potential arrives at a synapse, it produces a string of events which culminate in a stereotyped current across the synaptic membrane of the downstream neuron. In this model, the stereotyped transient current of a single synaptic input had a sharp rise, and an exponential decay with timescale of $\tau = (2\pi 70Hz)^{-1} = 2.3ms$ consistent with empirical measurement (Sabatini BL & Regehr WG (1996) Nature 384, 170-172.). The superposition of many of these synaptic input currents perturbs the trans-membrane difference in charge concentration between the inside and outside of the neuron (Connor JA & Stevens CF (1971) The Journal of Physiology 213, 1-19.). The new trans-membrane potential difference caused by this difference in charge concentration causes ohmic current, of charged ion species, through the dendritic membrane and the proximal soma. In this simulation, we approximate the timecourse of the dipole that gives rise to our potentials as the timecourse of this ohmic current. We created 2 minutes of 10kHz sampled simulated data for a single neuron with 6000 synaptic inputs, each with current influx delay timescale of order $\tau = (2\pi f_0)^{-1}$, and peak current magnitude randomly chosen on the interval from -1 to 1 (arbitrary units), for each synapse. Mean input action potential rates of 15, 30, and 60 $\frac{AP}{synapse*s}$.

We provide a basic simulation in order to demonstrate how this power-law scaling we observe experimentally might arise from neuronal processes at the most basic level. While this simulation is clearly an over-simplification, it may provide useful insight into how such power-of-2, integer, scale free processes might arise in cortical signals. In particular, it demonstrates how power-law spectra might directly reflect the aggregate, average firing rates of the *inputs* to a cortical population. Note that the model presented below only addresses the *time dependence* of simple processes in a single neuron, neglecting spatial summation across neurons and the form that the post-synaptic dipoles which produce the macroscopic potentials actually might take.

The simulation for the time dependence of the currents that produce these current dipole potentials consists of a single neuron with 3 basic elements which produce the time-course of the current dipole, and is illustrated in Fig 5 of the main text and Fig. S21:

Poisson distributed input action potentials: The first element is the generation of 6×10^3 input spike trains with Poisson-distributed spike arrival times times (Figs. 5C and S21) to simulate the presynaptic input.

Exponentially-decaying post-synaptic potential: Each input action potential is convolved with a post-synaptic, exponentially decaying, current shape as in Fig. S21. The convolved spike train is multiplied by a random scalar between -1 and 1 to reflect different signs and magnitude of different synapses. The exponential decay gives rise to one factor of $\frac{1}{f^2}$ in the $P \simeq \frac{1}{f^4}$ above f ~80Hz. At lower frequencies, τ produces a knee in the PSD, and in the model, produces a Lorentzian, $\frac{1}{1+(f/f_0)^2}$. The relation between f_0 and τ is then $\tau = (2\pi f_0)^{-1} = 2.3ms$. If a range of times, centered at $\tau = 2.3ms$, rather than a single value is used, the results of the simulation are similar, but the knee is not as sharp.

Temporal accumulation of synaptic current, and loss across the dendritic membrane: These post-synaptic current / action potential temporal convolutions are summed across synapses, and the difference in charge across the membrane, $*([Q]_{in} - [Q]_{out})$, accumulates over time, and is lost ohmically as leaky current through the dendritic membrane. The leakage current of this charge through the dendritic membrane is what we simulate as the time-dependence of the dendritic dipole (with time constant α^{-1} below).

$$\frac{\partial I(t)}{\partial t} = -\alpha I(t) + Q(t) \tag{8}$$

$$Q(t) = \sum_k s_k \; conv\,(\eta(\tau)\epsilon_k(t)) \tag{9}$$

Where we denote a series of delta functions reflecting the spike arrival times at synapse k as $\epsilon_k(t)$, the shape of the post-synaptic response as $\eta(\tau)$ (total length T), a random number on the interval from -1 to 1, as s_k, the decay timescale for dendritic current efflux as α, and the convolution operation $conv\,(a(\tau), b(t)) = \int_{-T/2}^{T/2} d\tau \; a(\tau)b(t+\tau)$ (and b is zero padded at the edges). $I(t)$is the simulated time dependence of our surface potential measurements, from which we calculate our simulated spectrum.

The PSD of these reproduces the static (fixation) and active (movement vs. rest) spectra that we observe reasonably well, and this is shown in figure 5 of the main text. There are many equivalent model processes that would generate the power-law type spectra that we measure experimentally. We believe that a common key feature of this model, and the other equivalent neural models, is that changes in the ***mean firing rate*** of input neurons are reflected by changes in scale-free, power-law, aspects of the spectra, where the shape is preserved, but the amplitude is not. The ability to capture non-oscillatory, broadband, change is a powerful tool, and is presented in a recent manuscript, *Decoupling the Cortical Power Spectrum Reveals Real-time Representation of Individual Finger Movements in Humans* by K.J. Miller et. al. in Journal of Neuroscience in 2009; **29**, 3132.
